# Nanoenabled Hyaluronic Acid-Based Topical Delivery of Kaempferol and Ferulic Acid: Chemomodulatory Potential Against DMBA-Croton Oil-Induced Skin Carcinogenesis in Male Swiss Albino Mice

**DOI:** 10.3390/pharmaceutics18080972

**Published:** 2026-08-07

**Authors:** Moulika Todaria, Rajendra Awasthi

**Affiliations:** 1 Department of Microbiology, School of Health Sciences and Technology, University of Petroleum and Energy Studies (UPES), Dehradun 248001, India; 2 Department of Pharmaceutical Sciences, School of Health Sciences and Technology, University of Petroleum and Energy Studies (UPES), Dehradun 248001, India

**Keywords:** chemopreventive, DMBA-croton oil, inflammation, topical delivery, skin cancer

## Abstract

**Background/Objectives**: Skin cancer is a major health concern worldwide and has led to the quest for safer and more effective topical chemopreventive agents. In this study, the in vivo efficacy of a hydrogel formulation containing kaempferol and ferulic acid-loaded nanoparticles was evaluated for the management of DMBA-croton oil-induced skin cancer in Swiss albino mice. **Methods**: Drug-loaded nanoparticles prepared via nanoprecipitation were incorporated into hyaluronic acid to obtain single-drug (FAPG, KMPG) and dual-drug-loaded (FKMG) hydrogel formulations. Skin tumors were induced via DMBA as an initiator followed by croton oil as a promoter, with tumor onset observed after a similar latency period of approximately 5–6 weeks in all carcinogen-exposed groups. Treatment was initiated after week 6 and continued until week 16. **Results**: The gel formulation had a skin-compatible pH and the desired rheological and spreadability properties. The dual drug-loaded hydrogel formulation had a more controlled and prolonged release profile. Compared with the negative and vehicle control groups, the FKMG-treated group presented a marked reduction in tumor incidence and tumor burden, along with improved body weight gain. Biochemical analysis revealed the restoration of antioxidant and enzyme activity in treated animals, particularly in animals treated with FKMG. Histopathological examination revealed near-normal skin architecture in FKMG-treated mice, indicating strong protective effects. Additionally, significant downregulation of TNF-α and IL-6 suggested effective suppression of tumor-promoting inflammatory pathways. **Conclusions**: Overall, the FKMG formulation exhibited superior chemopreventive efficacy compared with the FAPG and KMPG treatments, highlighting its potential as a promising topical therapeutic strategy against chemically induced skin carcinogenesis.

## 1. Introduction

Skin cancer represents a significant and potentially life-threatening public health concern, particularly among fair-skinned populations. The exposure of the skin to environmental carcinogens, ultraviolet (UV) radiation, and microbial agents constitutes major risk factors contributing to skin carcinogenesis. Clinically, skin cancer can be classified as melanoma skin cancer or nonmelanoma skin cancer. Epidemiological studies indicate a continuous increase in the incidence of both melanoma and nonmelanoma skin cancer worldwide [1]. Considering the growing prevalence of skin cancer and the significant adverse effects associated with conventional chemotherapeutic agents, there is an increasing demand for safer and more effective therapeutic alternatives. Natural products, particularly those derived from plants, have long been utilized in traditional medicine for disease prevention and treatment because of their wide availability, cost-effectiveness, and ability to modulate multiple cellular signaling pathways involved in disease progression [2]. The remarkable structural diversity of plant-derived compounds provides a valuable resource for the development of novel chemotherapeutic agents. This is further supported by pharmaceutical evidence indicating that among the 131 anticancer drugs approved between 1981 and 2014, approximately 85 were either natural products or directly derived from natural sources [3].

Ferulic acid (FA) is a derivative of caffeic acid and has been found in plants, fruit, and beverages such as coffee and beer. FA has been identified as an emerging antioxidant molecule with potent cytoprotective properties mediated by free radical scavenging and cellular stress response-activating properties [4]. The anticancer properties of FA are mediated by its ability to inhibit cell proliferation, induce apoptosis, inhibit invasion and migration of tumor cells, and enhance the effectiveness of chemoradiotherapy regimens. It may also modulate the immune system, induce autophagy, and suppress drug resistance in cancer cells [5]. Kaempferol (KMP), a tetrahydroxyflavone, is an aglycone flavonoid with a yellow pigment and is found in strawberries, tea, apples, and broccoli. It activates several signal transduction pathways that regulate the activity of several major cellular signal transduction molecules, thereby mediating their anti-inflammatory, antioxidant, antimicrobial, and neuroprotective properties [6]. Compared with conventional chemotherapeutic agents, KMP has been shown to have pharmacological properties that reduce the risk of chronic diseases, including cancer, with minimal adverse effects on normal cells [7]. KMP inhibits tumor cell proliferation by suppressing key signal transduction pathways, particularly the phosphoinositide 3-kinase/protein kinase B (PI3K/Akt) signaling pathway, inducing apoptosis, promoting cell cycle arrest at the G2/M phase, and modulating molecular markers associated with epithelial–mesenchymal transition [8]. Despite their wide range of pharmacological properties, the biomedical application of FA and KMP is restricted by their low water solubility, permeability, instability in alkaline solutions, extensive metabolism before systemic absorption, and poor oral bioavailability [9].

The dermal permeation of pharmaceutical agents can be improved through the application of nanotechnology. Nanocarrier-based delivery systems increase drug bioavailability by facilitating improved penetration and controlled release within the skin layers. Additionally, these systems can reduce the likelihood of skin irritation by enabling targeted delivery and minimizing the exposure of surrounding tissues to the active compound [10,11]. Poly(lactic–coglycolic) acid (PLGA) is a widely used synthetic biodegradable polymer approved by both the US Food and Drug Administration (FDA) and the European Medicines Agency (EMA) for targeted drug delivery applications. It is a copolymer composed of two monomers, lactic acid and glycolic acid, which undergo hydrolysis in the body to produce their respective monomers. Since these degradation products are naturally occurring metabolites, PLGA exhibits minimal toxicity [12]. PLGA) has been reported to be effective in topical drug delivery. Its extensive applications in drug delivery systems and medical devices have been well documented. It can encapsulate both hydrophilic and lipophilic drugs and is highly versatile for surface modification to promote drug delivery [13]. PLGA NPs are recognized as an important platform in cancer immunotherapy because of their dual function in targeted drug delivery and immune modulation. By encapsulating chemotherapeutic agents such as paclitaxel and vinorelbine, PLGA NPs improve the therapeutic efficacy of these drugs through their selective delivery to tumor tissues. This targeted delivery approach reduces systemic toxicity and minimizes damage to healthy tissues. In addition, the versatility of PLGA enables the encapsulation of a wide range of bioactive molecules, including cytokines, antigens, and other immunomodulatory agents, allowing precise regulation of the immune response [14].

Another method for developing novel polymer–drug formulations for topical application is the use of chemically cross-linked hydrogels. Three-dimensional, water-soluble polymer networks known as hydrogels can absorb significant volumes of biological fluids or water. The distinctive physical properties of chemically cross-linked hydrogels have gained significant attention in improving the therapeutic efficacy of payloads with few adverse effects [15]. The structure of hydrogels is easily modified to suit the needs of drug delivery systems by adjusting the density of cross-links present in the polymer network and the affinity of the hydrogels for the aqueous environment. Hydrogels can provide localized treatment with antineoplastic agents via topical application, thus avoiding the systemic toxicities associated with chemotherapy and the risk of infections associated with surgery [16].

The multistage model of skin carcinogenesis is commonly used to study the development of cancer. In this model, skin cancer is induced via DMBA and croton oil, which are used to damage DNA, and the development of cancer is observed over a short period of time. DMBA causes DNA mutation and oxidative stress, and croton oil causes chronic inflammatory responses, thus providing a microenvironment for tumor growth [17,18]. This study explored skin carcinogenesis induced by DMBA and its potential treatment with KMP-FA-loaded dual-delivery polymeric nanoparticle hydrogels. The parameters studied were tumor incidence, tumor burden, the spleen-to-body ratio, and biochemical parameters, including anti-inflammatory cytokines.

## 2. Materials and Methods

### 2.1. Materials

Hyaluronic acid (M.W: 403.31) was purchased from Yarrow Chem Products (Mumbai, India). Ashland Specialties Ltd., Mullingar, Ireland provided PLGA (Viatel^TM^ DLG 7502 E with a lactide: glycolide ratio of 75:25 and an inherent viscosity of 0.1–0.3 dL/g). Tween 80 was purchased from Avantor Performance Materials India Pvt. Ltd., Mumbai, India. Propylene glycol, glycerin, methylparaben, and propylparaben were obtained from Sisco Research Laboratories Pvt. Ltd., Maharashtra, India. DMBA and croton oil were purchased from Tokyo Chemical Industry Co. Ltd., Tokyo, Japan. Flonida^®^ cream (1%) was purchased from Menarini India Pvt. Ltd. Mumbai, India. Formalin was obtained from SRL Chemicals (Mumbai, India). Tris-HCl, glutathione, trichloroacetic acid, and DTNB 133 (5,5-dithiobis (2-nitrobenzoic acid)) were obtained from Sigma Chemical Co. in St. Louis, MO, USA.

### 2.2. Methods

#### 2.2.1. Formulation of a Hydrogel Containing Polymeric Nanoparticles

The formulation and primary characterization of KMP-FA coloaded polymeric nanoparticles (NPs) were detailed in our prior publication [19]. In brief, the nanoprecipitation technique was used to formulate KMP- and FA-loaded polymeric NPs. KMP (10 mg in 10 mL) and FA (10 mg in 10 mL) were first dissolved in ethanol, while PLGA (50 mg/20 mL) was dissolved in acetone to form the organic phase. The aqueous phase (250 mg/100 mL) included PVA (0.25%), which was used as a stabilizer. The organic phase containing PLGA and the drug was injected dropwise into the aqueous PVA solution with continuous sonication at 80 kHz via an ultrasonic probe sonicator (UP200Ht, Hielscher, Teltow, Germany). Sonication of the NP dispersion at 25 °C allowed evaporation of the organic solvent. KMP-loaded polymeric NPs and FA-loaded polymeric NPs were formulated via a similar process to study the effects of individual bioactive-loaded polymeric NPs and compare their in vivo effectiveness to that of dual-delivery polymeric NPs. Polymeric NPs (20 mL equivalent to 150 μg/mL of FA and 100 μg/mL of KMP) were mixed with hyaluronic acid (HA) gel through the cold hydration process. Briefly, in 20 mL of double-distilled water, NPs, 2% *w*/*v* HA, 5% *v*/*v* ethanol, 0.2% *w*/*w* methylparaben, and 0.1% *w*/*w* propylparaben were added. NPs and Tween 80 (0.25% *v*/*v*) were mixed with a HA gel base via a magnetic stirrer. The gel system was allowed to swell at 25 °C for 24 h. Propylene glycol (0.25%) and glycerin (0.25%) were added to the NP-containing hydrogel system [20]. The hydrogel formulations containing FA, KMP, and FA-KMP-loaded polymeric NPs are coded as FAPG, KMPG, and FKMG, respectively.

#### 2.2.2. Morphological Characterization

A field emission scanning electron microscope (FE-SEM; Apreo LoVac, FEI Company, Hillsboro, Oregon, USA) was used to analyze the surface morphology of the FKMG. Sample preparation was done by air-drying the FKMG gel on a glass slide. To provide sufficient conductivity, a thin layer of gold was coated on the dried sample using cathode evaporation. Micrographs were taken at an accelerating voltage of 5–15 kV.

#### 2.2.3. Determination of pH

A pH meter was used to record the pH of the gel formulations (FAPG, KMPG, and FKMG), the drug-free hydrogel (PPG), and the simple gel without NPs (PNG). Briefly, 1 g of sample was dissolved in 25 mL of distilled water, and the electrode was immersed in the gel sample until a consistent reading was obtained. The pH of each formulation was recorded in triplicate [21].

#### 2.2.4. Rheological Study

An Anton Paar C-LTD80/QC rheometer (Graz, Austria) was used to test the viscosity and flow characteristics of the FAPG, KMPG, FKMG, PPG, and PNG samples (10 mL) at a regulated phase–shear pressure. After the hydrogel sample was placed into the sample holder, a shear rate ranging from 0.1 to 1000 s^−1^ was applied. Rheoplus/32 (version 3.62) software was used to record viscosity and shear stress. The obtained data were fitted according to the Bingham plastic (τ = τ_0B_ + ηẏ^n^), power law (τ = k ˙*γ*^n^), and Herschel–Bulkley (τ = τ_0H_ + k_H_ ˙*γ*^n^) models. where τ is the shear stress, k is the consistency coefficient, γ̇ is the shear rate, n is the flow behavior index indicating the nature of flow behavior, τ_0B_ is the Bingham yield stress, η_B_ is the Bingham plastic viscosity, τ_0H_ is the Herschel–Bulkley yield stress, and k_H_ is the Herschel–Bulkley consistency coefficient [22].

#### 2.2.5. Spreadability Analysis

The spreadability of the FAPG, KMPG, FKMG, PPG, and PNG hydrogels was measured via the parallel plate method. Briefly, 0.5 g of each sample was placed at the center of a clean glass slide. Another glass slide was placed concentrically on top of it. The initial diameter of the circular region formed by the hydrogel sample was measured. A weight (200 g) was placed on the top glass slide for one minute to allow the gel to spread. The following equation was used to calculate spreadability [23]:

$$Spreadability=\frac{\pi d^{2}}{4}$$

where d is the average diameter of the spread gel.

#### 2.2.6. In Vitro Drug Release Study and Kinetic Modeling

To investigate the in vitro release of KMP and FA from FAPG, KMPG, and FKMG, the activated dialysis membrane containing the hydrogel sample (equivalent to 5 mg of KMP and 5 mg of FA) was submerged into a beaker containing 200 mL of phosphate-buffered solution (PBS, pH 6.8). The temperature of the dissolution medium was maintained at 37 ± 0.5 °C, and the mixture was stirred at 100 rpm via a magnetic stirrer. At specific intervals, 5 mL of the solution was collected and replaced with an equal volume of fresh PBS. The released amounts of KMP and FA were estimated via a UV–visible spectrophotometer (UV-1900, Shimadzu, Kyoto, Japan) at 366 nm and 310 nm, respectively. Release was analyzed via zero-order, first-order, Higuchi equation, and Korsmeyer–Peppas models [24].

#### 2.2.7. In Vivo Evaluation of PLGA NP-Loaded Gel

##### Animals

The animal experimental protocol was approved by the Institutional Animal Ethics Committee of UPES Dehradun (UPES/IAEC/2024/5/07; dated 13 December 2024). Thirty-five male Swiss albino mice (4–6 weeks of age; 30–35 g) were used in this experiment. Animal handling and maintenance were performed in accordance with the guidelines of CCSEA, New Delhi, India. All experiments complied with the principles of the 3Rs (Replacement, Reduction, and Refinement) and were designed to minimize animal suffering. Animal care and use were consistent with internationally accepted standards, including the U.K. Animals (Scientific Procedures) Act 1986, Directive 2010/63/EU, and the Guide for the Care and Use of Laboratory Animals (NIH Publications No. 8023, revised 1978). The animals were housed in polypropylene cages and maintained under controlled laboratory conditions (26 ± 2 °C and 55 ± 5% RH; 12 h light/dark cycle) with free access to standard food and water during the experiment.

##### Induction of Skin Tumors

The animals were divided into seven experimental groups (n = 5) (Table 1). Using DMBA-containing croton oil, skin tumors were induced in male Swiss albino mice. Before 48 h of DMBA application, the dorsal hairs were removed via an electric clipper. Depilatory cream (Reckitt Benckiser India Ltd., Gurugram, Haryana, India) was applied to the dorsal skin. Once daily for two consecutive weeks with a 7-day interval between applications, 25 µg of DMBA was dissolved in 100 µL of acetone and topically applied to each animal on shaved dorsal skin. Tumor promotion was initiated from the third week onward via the addition of croton oil (1% *v*/*v* in acetone). Croton oil was applied topically at a dose of 200 µL per animal three times per week until the completion of the study at 16 weeks. At the end of the 16th week, the experiment was terminated. The animals were euthanized, and the skin and tumors were dissected for analysis [25].

##### Assessment of Tumor Development

The mice were observed throughout the study for the appearance of tumors. The latency period, percentage tumor incidence, and tumor burden were determined. For data analysis, only tumors exceeding 1 mm in diameter were included. The latency period corresponded to the time taken for the first tumor to appear. The percentage of tumor incidence was calculated as the ratio of tumor-bearing mice to the total number of mice in each group × 100. The tumor burden was defined as the total number of tumors divided by the number of tumor-bearing animals in the group [1].

##### Histopathological Studies

At the end of the experiment, the animals were sacrificed, and fresh samples of the skin, tumor, and spleen of each animal were collected and fixed in neutral buffered formalin (10%). Paraffin-embedded skin tissues were sectioned into thin slices (3–5 µm) via a microtome, and the sections were stained with hematoxylin and eosin (H&E). A light microscope was used to view the stained sections, and photomicrographs of the experimental and control groups were captured [26].

##### Biochemical Estimation

Excised skin tissues from the mice in each experimental group were washed thoroughly with ice-cold phosphate-buffered saline (pH 6.8). The tissues were homogenized, and the homogenate was centrifuged at 1500 rpm for 5 min. The resulting supernatant was collected for biochemical analyses of GSH, superoxide dismutase (SOD), catalase, lipid peroxidation, and total protein content.

• Estimation of GSH S-transferase activity

The concentration of the protein present in the sample was determined via the Bradford method [27]. The enzyme activity was represented as units per milligram of protein. The reaction mixture contained 10 µL of oxidized glutathione, 10 µL of NADPH, 70 µL of potassium phosphate buffer (1 M), and 10 µL of the sample. The mixture was prepared in triplicate, with blanks prepared in duplicate without the sample. The decrease in absorbance at 340 nm was measured with a microplate reader (iMark^TM^, Bio-Rad, USA). Readings were taken every min for 10 min. The GSH reductase activity was determined via the following formula:

$$GR(U/mL)=\frac{\Delta OD\;\times \;V}{\varepsilon *v}\times df$$

where ΔOD is the change in absorbance per minute at 340 nm, V is the total reaction volume, v is the sample volume, ε is the molar extinction coefficient of NADPH (0.00622 µM^−1^ cm^−1^), df is the dilution factor, and the final activity is expressed as units per milligram of protein.

• Estimation of lipid peroxidation

Lipid peroxidation was quantified via a thiobarbituric acid-reactive substances (TBARS) assay to measure malondialdehyde (MDA) levels [28]. For the TBARS assay, 50 μL of sample was added to 150 μL of trichloroacetic acid and centrifuged at 2500 rpm for 5 min to precipitate the proteins. The resulting supernatant was added to 150 μL of the thiobarbituric acid reagent and incubated at 80 °C for 15 min. After incubation, the mixture was analyzed at 532 nm via a UV–vis spectrophotometer (Shimadzu, Kyoto, Japan). The concentration of MDA was determined via the following extinction coefficient: 1.56 × 10^5^ M^−1^ cm^−1^.

• Estimation of superoxide dismutase activity

The Assay of SOD (Cat No. KEA006A) is based on the inhibition of the formation of GSH Phenazine-Metho-nitro blue tetrazolium formazan, blue color crystals [29]. The reagents and samples were first equilibrated at 37 °C. The enzyme standard solution of 100 U/mL was made by diluting 10 µL of enzyme with 90 µL of buffer solution. SOD standards were also made at concentrations ranging from 6.25–100 U/mL. The test samples, blanks, standards, and controls were pipetted into a 96-well microtiter plate. The reaction mixture was made of 1.2 mL potassium pyrophosphate buffer (0.025 M, pH 8.3), 1 mL phenazine methosulfate (186 µM), and nitroblue tetrazolium (300 µM). Crude enzyme extracts (0.05 mL) and 1× PBS (0.05 mL) were added, and the final volume was made up to 8 mL using distilled water. To make the volume of the reaction up to 8 mL, 1.15 mL dH_2_O was added, then the reaction was initiated by adding 0.2 mL GSH (780 µM), and the absorbance was recorded at 570 nm via a microplate reader (iMark^TM^, Bio-Rad, USA). The activity of SOD was determined via a standard curve and was expressed as U/mL.

• Estimation of catalase activity

Catalase is a key antioxidant enzyme that protects cells from oxidative damage by catalyzing the decomposition of hydrogen peroxide (H_2_O_2_) into water and molecular oxygen. In the present study, catalase activity was determined indirectly by measuring the residual H_2_O_2_ remaining after enzymatic decomposition using the ammonium molybdate colorimetric assay (Cat No. KEA005A) [30]. The enzyme solution (100 U/mL) was prepared by diluting 10 µL of catalase with 90 µL of buffer, and the peroxide solution was diluted by adding 25 µL of peroxide stock solution to 10 mL of buffer. The assay was performed on a 96-well plate with an appropriate blank, an enzyme control, and test samples, followed by incubation at 37 °C for 5 min. The reaction was stopped with ammonium molybdate (32.4 mM in 25 mM H_2_SO_4_) and incubated for 2 min, after which the absorbance was recorded at 415 nm via a microplate reader (iMark^TM^, Bio-Rad, USA). The catalase activity was expressed as U/mL.

$$Catalase\;activity(U/mL)=\frac{2.303}{t}\times \left(log\frac{S^{{}^{\circ}}}{S-M}\right)\times \frac{Vt}{Vs}$$

where t represents the reaction time, S° represents the absorbance of the enzyme control, S represents the absorbance of the test sample, M represents the absorbance of the control sample (correction factor), Vt represents the total volume of reagents in the reaction mixture, and Vs represents the volume of sample used.

• Estimation of total protein content

The total protein content was estimated via the Bradford method [31]. Briefly, bovine serum albumin standards (200–1000 µg/mL) were prepared for the standard curve. In a 96-well plate, 5 µL of each standard or sample was mixed with 200 µL of Bradford reagent. The plate was incubated at room temperature for 15 min, and the absorbance was measured at 595 nm. The protein concentration was determined from the standard curve and expressed as µg protein/mg tissue.

##### Estimation of Proinflammatory Cytokines

Tumor necrosis factor-alpha (TNF-α) and interleukin-6 (IL-6) levels were quantified via commercially available ELISA kits (Mouse TNF-α ELISA, Product No. W20852; Mouse IL-6 ELISA, Product No. W20188). The wells were filled with 50 µL of standards and samples, followed by 100 µL of HRP-labeled antibodies and an hour of incubation at 37 °C. Following incubation, wash buffer was used to rinse the wells five times. Each well was filled with 50 µL of tetramethylbenzidine and 50 µL of hydrogen peroxide solution. The last incubation was carried out in the dark for fifteen minutes at 37 °C. Stop solution (50 µL) was added to halt the enzymatic process. The absorbance was measured at 450 nm.

#### 2.2.8. Stability Study of Optimized PLGA Nanoparticles

The optimized NPs were stored in tightly sealed glass vials under two storage conditions: refrigerated (5 ± 3 °C) and ambient conditions (25 ± 2 °C/60 ± 5% RH) for a period of 6 months. At the end of the storage period, particle size and polydispersity index (PDI) were determined using a ZS Malvern Zetasizer (Malvern, Worcestershire, United Kingdom). Zeta potential was determined using a Zetasizer (ZTS1240, Malvern Panalytical, Worcestershire, United Kingdom) [32]. The results were compared with those of the freshly prepared optimized NPs as reported earlier to assess formulation stability [19].

#### 2.2.9. Statistical Analysis

The results are expressed as the mean ± standard error of the mean (SEM). The significance of differences between all gel formulations in the spreadability study was analyzed by one-way analysis of variance (ANOVA) followed by Tukey’s multiple comparison test. The significance of differences between the negative control group and the other groups was analyzed by one-way ANOVA followed by Dunnett’s multiple comparison test (ns, *p* ≥ 0.12; * *p* < 0.033; ** *p* < 0.002; *** *p* < 0.001). Statistical analysis was performed via GraphPad Prism 10 trial version software.

## 3. Results

### 3.1. Preparation of Nanoparticle-Loaded Hyaluronic Acid Hydrogel

FA- and KMP-loaded PLGA NPs [19] were embedded into the HA hydrogel matrix through the cold hydration process. The prepared hydrogel formulations (FAPG, KMPG, and FKMG) were homogeneous and free from particle aggregation in the hydrogel matrix. The process of cold hydration permitted slow swelling of the HA polymer chain, leading to the formation of a stable 3-D physically cross-linked network that retained NPs inside the hydrogel matrix without compromising the integrity of NPs.

HA has been used as the hydrogel matrix owing to its superior biocompatibility, biodegradability, non-immunogenic properties, and natural attraction to skin tissues. It is a hygroscopic substance that can attract 1000-times water molecules of its own weight. HA possesses a remarkable capacity to bind and retain water molecules, enabling it to maintain skin hydration by preserving moisture within both the stratum corneum and the dermis. Owing to its hygroscopic properties, HA functions as a natural humectant, facilitating the movement and retention of water from the dermis to the epidermis. Within the dermal extracellular matrix, HA plays a critical role in regulating tissue hydration, osmotic balance, and ion transport. It also contributes to the structural organization of the extracellular matrix by forming a hydrated network that acts as a molecular sieve, selectively restricting the diffusion of certain molecules while preserving tissue integrity through electrostatic interactions [33]. The excellent biocompatibility, biodegradability, and non-immunogenic nature of HA, together with its high affinity for the CD44 receptor, have made it an attractive biomaterial for pharmaceutical applications. CD44-mediated internalization further enhances the potential of HA for targeted therapeutic delivery [34]. The interaction between HA and CD44 also plays a significant role in tumor progression and metastasis. HA-CD44 signaling modulates the binding and activation of cytokines, chemokines, and growth factors with their respective receptors, thereby promoting multiple oncogenic processes, including cell proliferation, differentiation, migration, and angiogenesis. Collectively, these signaling events facilitate tumor invasion and metastatic dissemination [35].

Tween 80 was incorporated as a nonionic surfactant to facilitate uniform dispersion of NPs by reducing interfacial tension and minimizing particle aggregation, thereby enhancing formulation stability. Propylene glycol served as a co-solvent and penetration enhancer, promoting transdermal diffusion of phytoconstituents by increasing hydration of the stratum corneum and consequently improving skin permeability. Glycerine functioned as a humectant, maintaining the moisture content of the hydrogel matrix and enhancing its flexibility and spreadability. Methylparaben and propylparaben were incorporated as antimicrobial preservatives to ensure the microbiological stability of the formulation throughout storage.

### 3.2. Morphology

**Figure 1:**
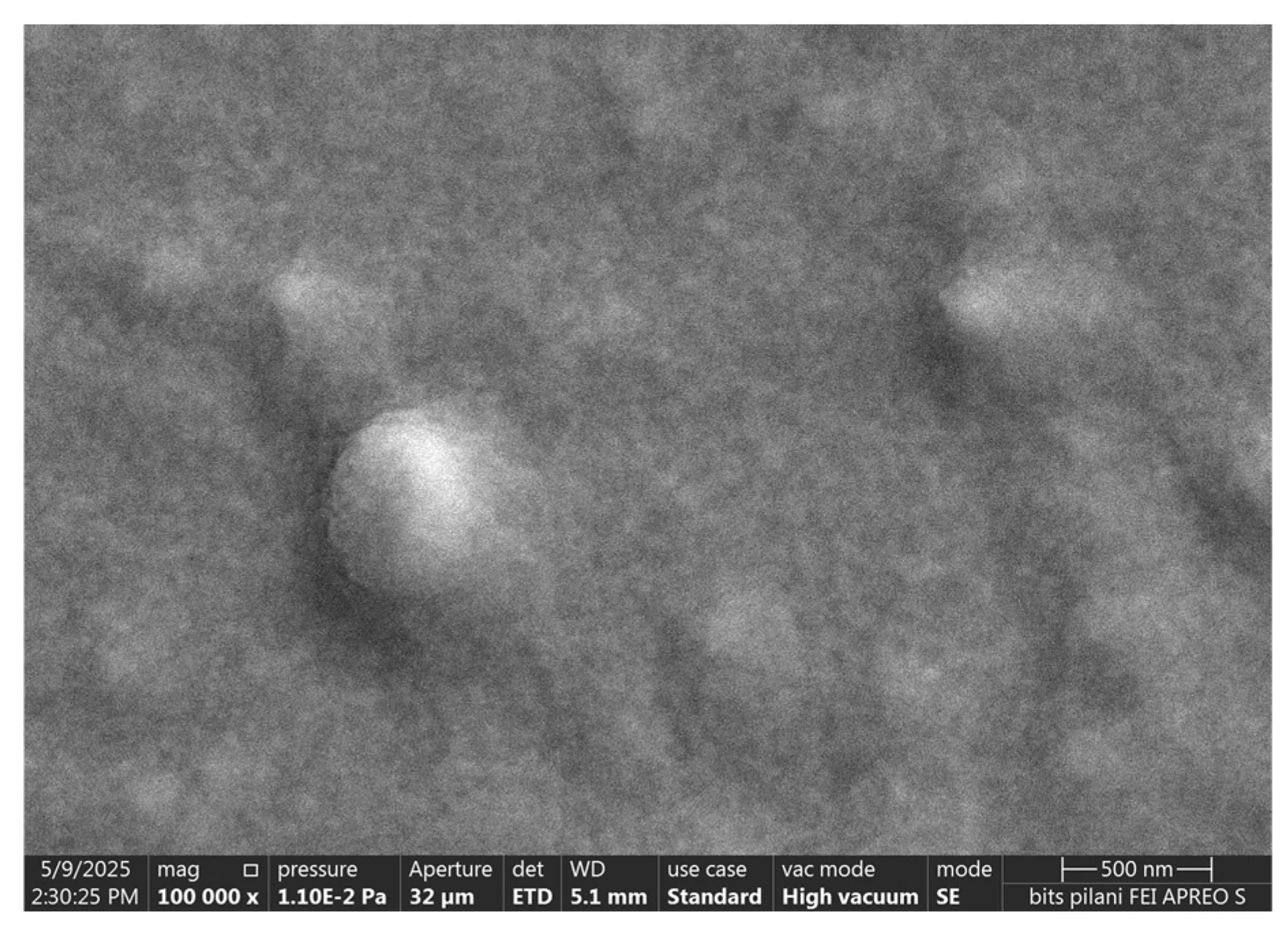
FE-SEM image of the FKMG hydrogel.

The FE-SEM micrograph of hydrogel formulation (FKMG) revealed a dense and continuous hydrogel matrix with a uniformly distributed nanosized particulate structure (Figure 1). NPs appeared as nearly spherical protrusions embedded within the hyaluronic acid network, indicating successful incorporation of NPs without obvious phase separation. The hydrogel exhibited a relatively smooth and compact surface with slight surface undulations, which are characteristic of hydrated polymeric matrices after drying. No large aggregates, cracks, or structural collapse were observed, suggesting good compatibility between the PLGA NPs and the hyaluronic acid matrix.

### 3.3. Determination of pH

The drug-free hydrogel (PPG) and simple gel without NPs (PNG) had pH values of 5.1 ± 0.6 and 5.4 ± 0.4, respectively. The drug-loaded NP hydrogel formulation (FKMG) had a pH of 5.5 ± 0.9 and was homogeneous. The pH values of the FAPG and KMPG formulations were 5.2 ± 1.2 and 5.6 ± 0.8, respectively. Both formulations had a homogeneous appearance. There was no effect on the pH of the hydrogel due to the loading of the drugs.

### 3.4. Rheological Behavior

**Figure 2:**
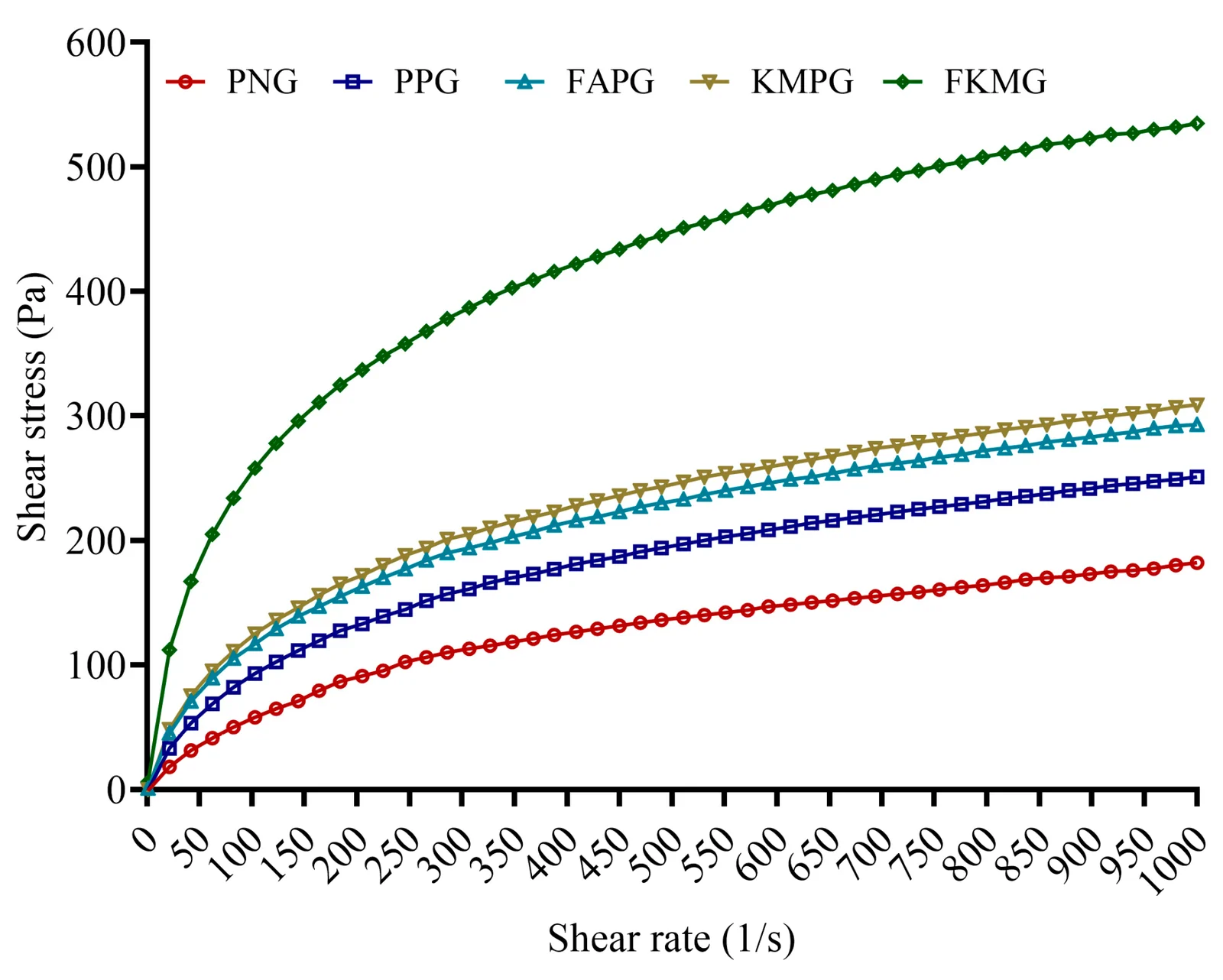
Rheological characterization shows shear stress vs. shear rate for NP-loaded hydrogels (FAPG, KMPG, FKMG), drug-free hydrogels (PPGs), and plain gels without NPs (PNG).

**Figure 3:**
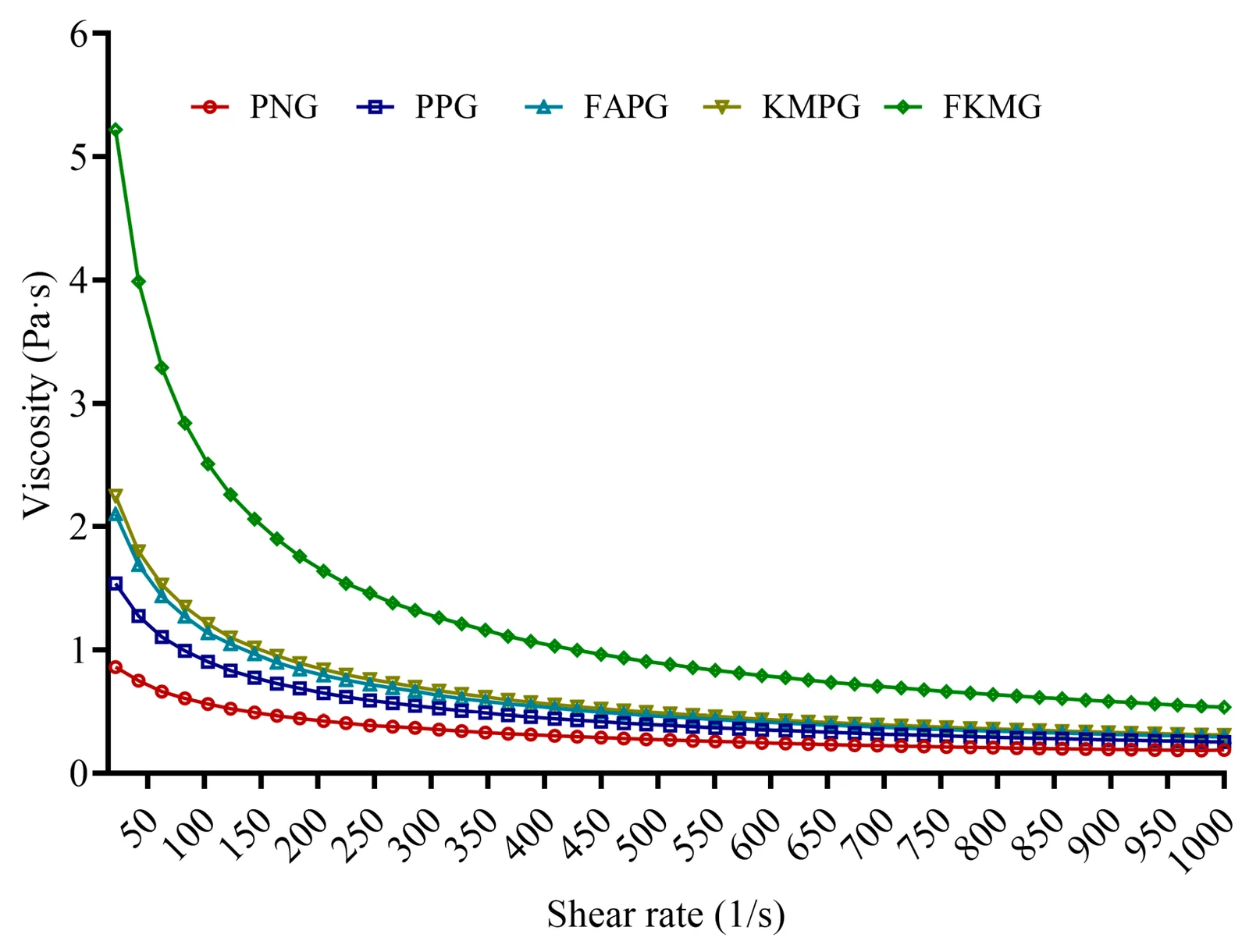
Time-dependent viscosity profiles of drug-loaded polymeric NP hydrogel formulations (FAPG, KMPG, and FKMG), drug-free hydrogels (PPGs), and plain gels without polymeric NPs (PNG).

The rheological properties of PNG, PPG, FAPG, KMPG, and FKMG are unique in terms of their flow behavior and structural arrangement. All the samples exhibited nonlinear relationships between shear stress and shear rate (0–1000 s^−1^). Among the samples, the FKMG sample presented the highest shear stress over the entire range of shear rates, reaching 535 Pa at 1000 s^−1^. The KMPG and FAPG formulations present intermediate values of shear stress, whereas the PPG and PNG formulations present relatively lower values (Figure 2). All the formulations exhibited a marked decrease in viscosity with increasing shear rate, confirming their non-Newtonian, pseudoplastic (shear-thinning) behavior (Figure 3). This behavior is characteristic of structured gel systems, where the applied shear progressively disrupts the internal network, resulting in reduced resistance to flow. At low shear rates, all formulations presented relatively high viscosities, indicating the presence of an intact and well-organized three-dimensional network. As the shear rate increased, a rapid decrease in viscosity was initially observed, followed by a more gradual decrease at higher shear rates. This two-stage behavior suggests the sequential breakdown of weaker intermolecular interactions, followed by the alignment and disentanglement of polymer chains in the direction of flow. HA hydrogel containing free FA and KMP, as well as the formulation incorporating empty PLGA NPs, exhibited rheological properties comparable to those of the plain hydrogel. This finding suggests that the incorporation of either free phytochemicals or unloaded NPs did not significantly alter the viscoelastic network of the HA matrix. The negligible change observed with the free drugs may be attributed to their presence as low-molecular-weight molecules, which are unlikely to substantially affect polymer chain entanglement or the overall hydrogel structure at the concentrations used. Similarly, blank PLGA NPs did not produce any measurable change in the flow behaviour of the hydrogel, indicating limited interaction with the HA polymer network under the present experimental conditions [36]. Among all the formulations, FKMG showed the highest viscosity, yield stress, and consistency coefficient, indicating the formation of a stronger gel structure. This improvement in rheological properties may be due to the incorporation of drug-loaded PLGA NPs within the HA matrix. Their presence is likely to influence the arrangement of the polymer chains and reduce their mobility, resulting in a gel that offers greater resistance to flow and deformation. Despite the increase in viscosity, the formulation maintained its shear-thinning behaviour, allowing it to spread easily under applied shear while remaining stable after application [37]. In contrast, the PNG formulation displayed a comparatively lower viscosity, indicating a less structured system. At higher shear rates, the viscosity of all formulations approached a near-constant region, indicating reduced sensitivity to further increases in shear and possible orientation of the polymer chains along the flow direction. However, variations in temperature may alter rheological behavior and other physicochemical attributes, potentially affecting product performance. Therefore, further investigations across a broader temperature range are warranted to establish the temperature-dependent rheological behavior of the formulations.

The rheological properties of the developed hydrogel formulations were evaluated via the Bingham model, power law, and Herschel–Bulkley model (Table 2). The flow behavior index (n), obtained from both the power law and Herschel–Bulkley models, was found to be less than one (0.45–0.66) for all formulations, confirming the pseudoplastic (shear-thinning) nature. This behavior is particularly desirable for topical drug delivery systems, as it ensures high viscosity at rest for improved retention at the application site while allowing a reduction in viscosity under shear, thereby facilitating ease of spreading. The yield stress (τ_0_) values obtained from the Herschel–Bulkley model ranged from 17.71 Pa (PNG) to 110.76 Pa (FKMG), indicating a progressive increase in structural strength across the formulations. In contrast, the Bingham model produced comparatively higher τ_0_ values (52.39–232.38 Pa). The Herschel–Bulkley model provided a more accurate representation of the nonlinear rheological behavior of the hydrogel systems. A clear trend was observed in the rheological parameters, with the yield stress (τ_0_) and consistency index (K) increasing in the order PNG < PPG < FAPG < KMPG < FKMG, indicating the formation of progressively stronger and more structured gel networks. The highest values were observed for FKMG (τ_0_ = 110.76 Pa; K = 20 Pa·s^n^), suggesting enhanced intermolecular interactions and network formation, likely due to the combined influence of the polymer concentration and active component incorporation. The flow behavior index (n) showed a decreasing trend, with the FKMG exhibiting the lowest value (0.45), indicating pronounced shear-thinning behavior. This combination of high τ_0_, high K, and low n suggests that FKMG possesses excellent structural integrity at rest along with superior spreadability under shear. Among the evaluated models, the Herschel–Bulkley model demonstrated the best fit, with R^2^ values ranging from 0.957 to 0.9798, confirming its suitability for describing the complex nonlinear rheological characteristics of the formulations.

### 3.5. Spreadability

**Figure 4:**
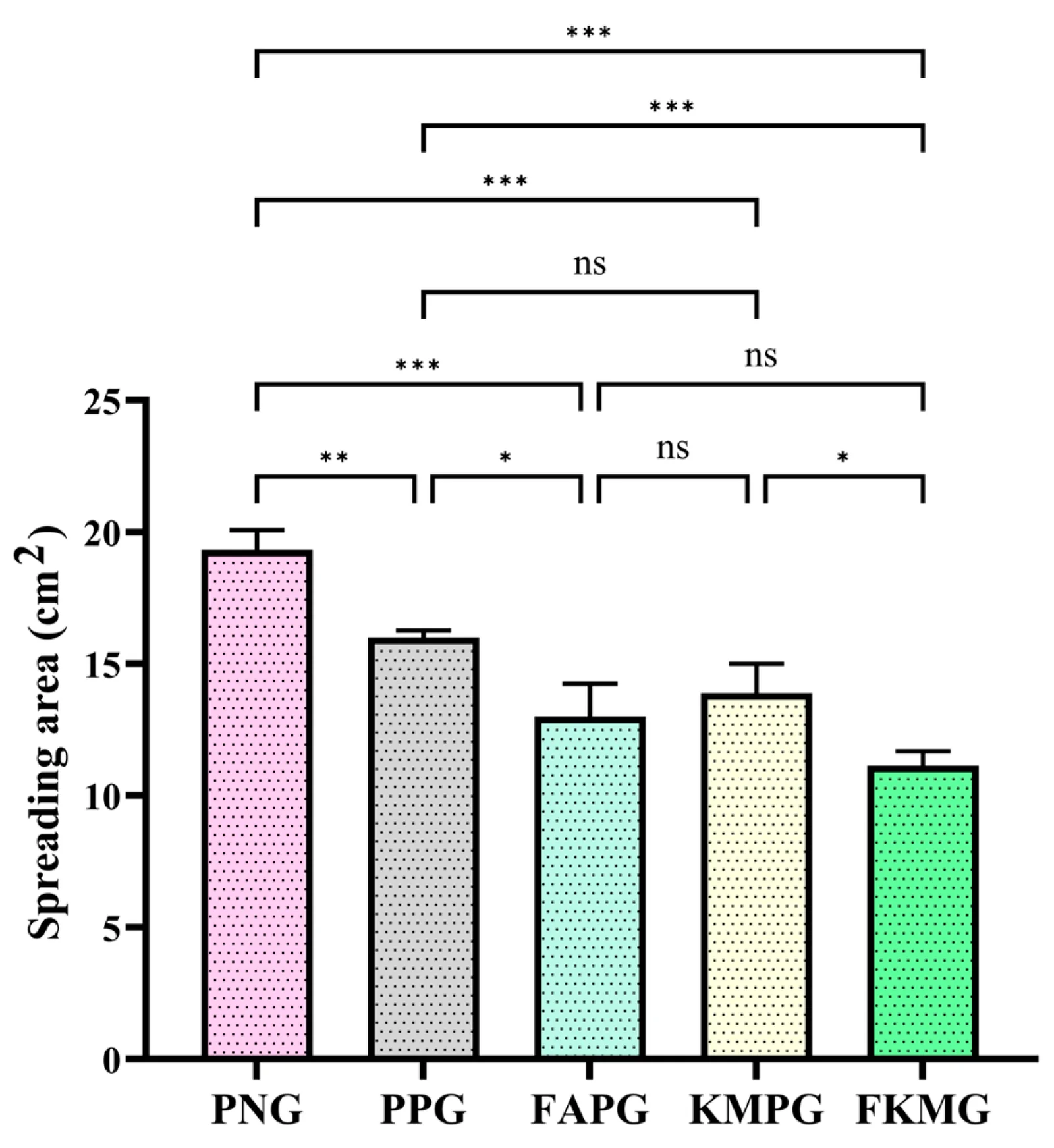
Determination of the spreadability of NP-loaded hydrogels (FAPG, KMPG, FKMG), drug-free hydrogel (PPG), and plain gels without NPs (PNG) (mean ± SD, n = 3). Statistical significance was assessed by one-way ANOVA followed by Tukey’s post hoc multiple comparison test (ns, *p* ≥ 0.12; * *p* < 0.033; ** *p* < 0.002; *** *p* < 0.001).

The spreadability of FAPG, KMPG, FKMG, PPG, and PNG is represented in Figure 4. With the application of 200 g of weight, the PNG formulation resulted in the highest spreadability (19.3 ± 0.75 cm^2^), which was significantly greater than that of the PPG (16 ± 0.26 cm^2^), FAPG (13 ± 1.24 cm^2^), KMPG (13.89 ± 1.11 cm^2^), and FKMG (11.13 ± 0.55 cm^2^) formulations. Spreadability decreased after the addition of bioactive compounds due to the increased viscosity of the polymeric gel. There was a significant difference in spreadability for PNG and PPG compared with FKMG. No significant difference was found between the spreadability of FAPG and KMPG.

### 3.6. In Vitro Drug Release Study and Kinetic Model Interpretation

**Figure 5:**
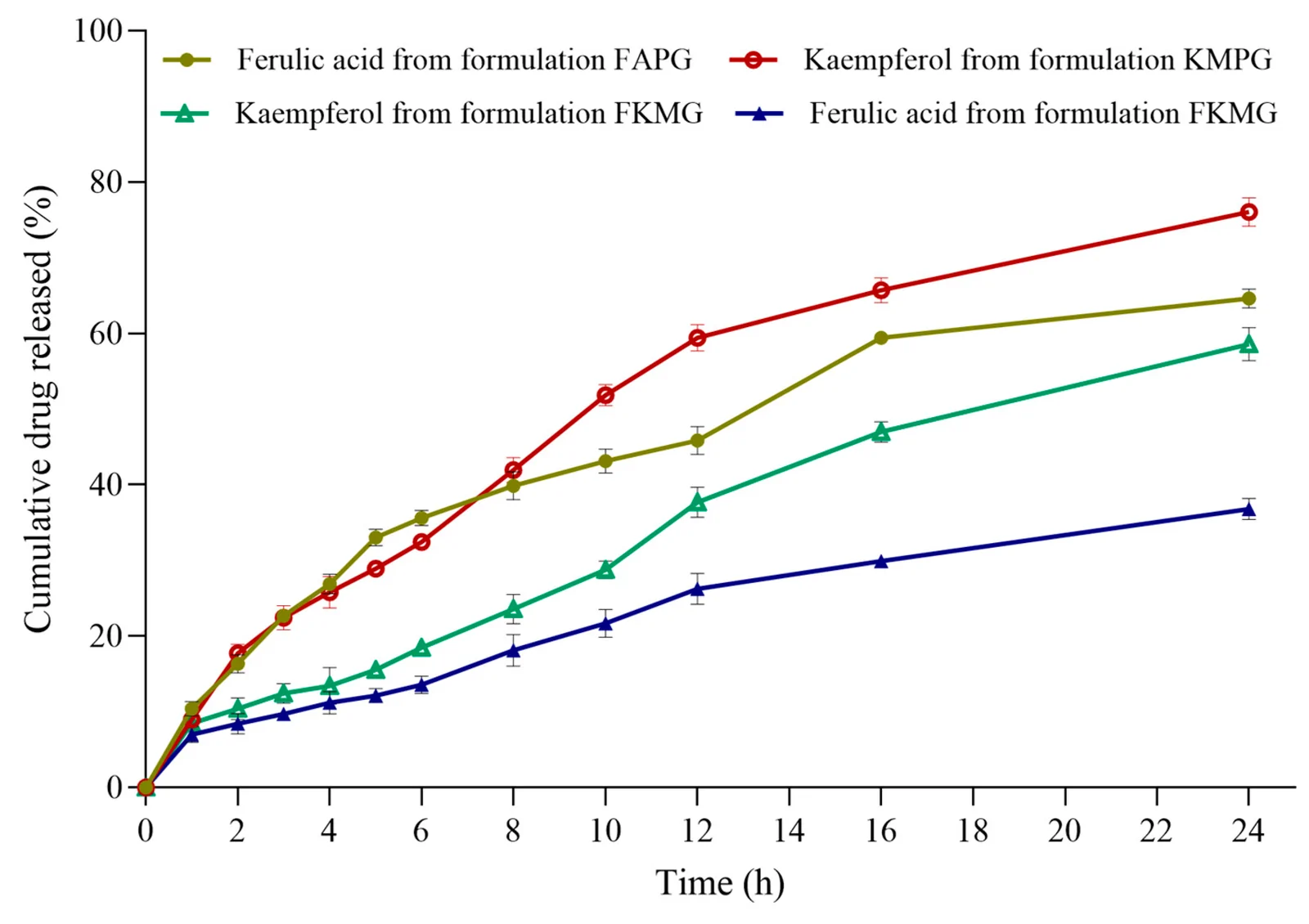
In vitro cumulative release of KMP and FA from the FAPG, KMPG, and FKMG hydrogels was determined in a phosphate-buffered solution (pH 6.8) and is expressed as the mean ± SD (n = 3).

KMP release from the KMPG formulation was 9.0 ± 0.85% in the initial hour, which gradually increased to 59.42 ± 1.41% by the end of 12 h and to 76.08 ± 1.90% by the end of 24 h. Similarly, the release of FA from the FAPG formulation was 10.40 ± 0.98% by the end of the initial hour, which gradually increased to 45.88 ± 1.86% by the end of 12 h and to 64.64 ± 1.25% by the end of 24 h. However, the release profile of the dual drug-loaded formulation was more controlled than that of the single drug-loaded formulation. This may be attributed to alterations in the polymer network structure induced by the co-loading of the drugs, which can modulate diffusional pathways and facilitate controlled drug release through the hydrated polymeric matrix. In the initial hour, the cumulative release of the drug from the formulation FKMG was found to be 7.00 ± 1.05% for FA and 8.50 ± 1.05% for KMP. During the initial release period (1–6 h), the cumulative release of the drug from FKMG gradually increased to 13.59 ± 1.12% for FA and 18.50 ± 0.70% for KMP. At the end of 12 h, the drug release from FKMG was 26.25 ± 2.05% for FA and 37.71 ± 2.00% for KMP. At the end of 24 h, drug release from FKMG gradually increased to 36.83 ± 1.40% for FA and 58.60 ± 2.20% for KMP (Figure 5). The greater release of KMP may be due to its higher solubility and higher affinity for the polymer matrix.

To understand the release mechanism, the dissolution data were analyzed via zero-order, first-order, Higuchi’s model, and Korsmeyer–Peppas model (Table 3). The first-order model showed the highest correlation for both bioactive compounds, indicating concentration-dependent release behavior. According to this hypothesis, drug release decreases over time because of the decrease in drug concentration in the polymer matrix. The Korsmeyer–Peppas model also showed good correlation. The calculated values of the release exponent were greater than 0.45. This confirmed that the release of the drug from the spherical PLGA NPs was anomalous (non-Fickian), implying a combined diffusion–relaxation process.

### 3.7. Effect of Treatments on Tumor Development in DMBA-Induced Swiss Albino Mice

**Figure 6:**
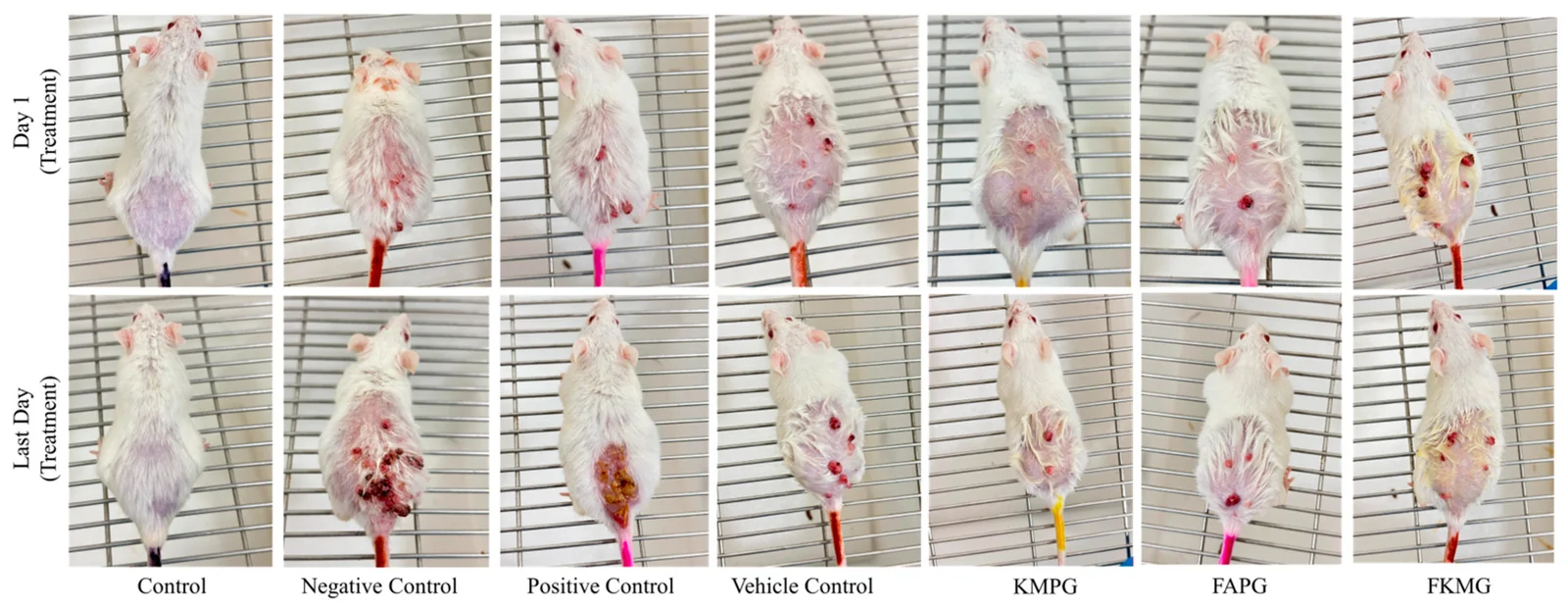
Progressive morphological alterations in tumors of the DMBA-croton oil-induced skin carcinogenesis model in male Swiss albino mice from week 6 to week 16.

DMBA treatment followed by three weekly administrations of croton oil was given to promote tumor formation. Tumor development began at approximately weeks 5–6 in all the DMBA-exposed groups (except the normal control), indicating a similar latency period of approximately 5–6 weeks across all the groups. No treatment was given during this initial phase; therefore, early tumor appearance reflects the direct carcinogenic effect of DMBA and croton oil. Therapeutic interventions were initiated after week 6 and continued until week 16, after which clear differences in tumor burden were observed among the treatment groups compared with the negative and vehicle control groups (Figure 6).

To further evaluate the therapeutic significance of the developed formulation, FKMG was compared with a commercially available 1% Flonida cream used as the positive control. The antitumor activity of Flonida is primarily mediated through inhibition of thymidylate synthase, resulting in suppression of DNA synthesis and apoptosis of rapidly proliferating cells [38,39]. The 1% formulation was selected instead of a 5% preparation because the aim of this study was to compare the investigational formulation with a commercially available fluorouracil product marketed in India. The use of a marketed formulation also improves the translational relevance of the study by reflecting current clinical practice in the local setting. Consistent with its pharmacological action, Flonida showed greater efficacy for certain tumor-related parameters than the test formulations. However, despite its anti-tumor activity, gross examination of the Flonida-treated animals revealed severe local skin toxicity, including extensive ulceration, marked tissue erosion, inflammatory exudate with purulent discharge, and complete absence of hair regrowth at the treated site. These findings indicate that effective tumor suppression was accompanied by substantial damage to the surrounding normal tissue.

The severe local tissue damage observed in the Flonida-treated group is consistent with the known adverse effects of topical 5-fluorouracil, which commonly include erythema, ulceration, crusting, delayed wound healing, and secondary infection during prolonged treatment [40]. In contrast, animals treated with FKMG showed progressive healing of the skin, preservation of normal tissue architecture, and visible hair regrowth, with no evidence of severe ulceration or purulent lesions. These findings indicate that the NP-loaded hydrogel achieved effective chemoprotective activity while exhibiting markedly better local tolerability than conventional topical chemotherapy.

### 3.8. Effect of Treatments on Body Weight and Tumor Parameters in DMBA-Induced Swiss Albino Mice

**Figure 7:**
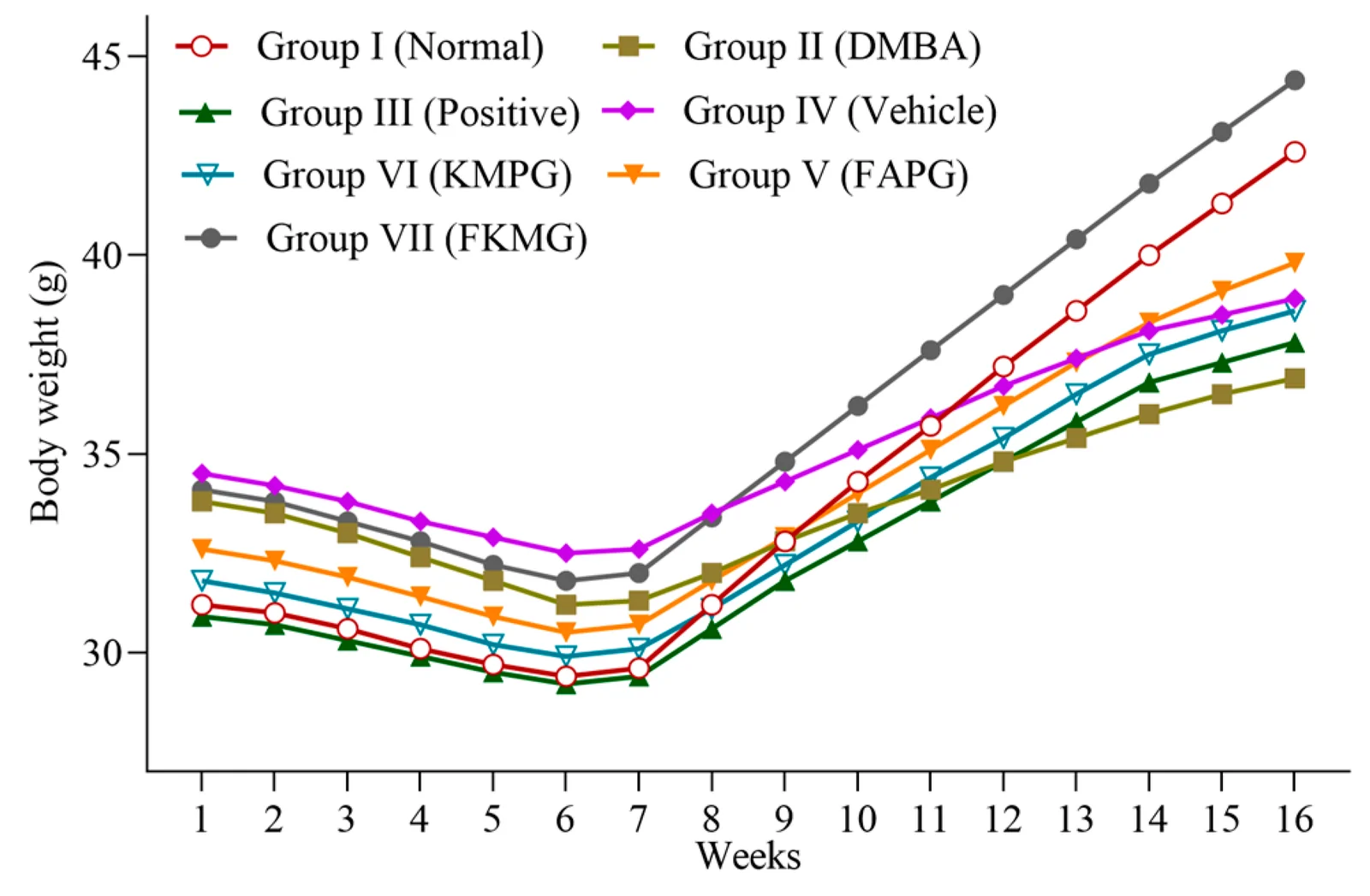
Effects of various treatments on body weight from week 1 to week 16.

Changes in body weight and tumor development were evaluated in DMBA-induced Swiss albino mice after 16 weeks of treatment to determine the effects of carcinogen exposure and the protective efficacy of the different experimental groups (Table 4). Body weight is an important indicator of general health and systemic toxicity in experimental animals. The data were recorded weekly from week 1 to week 16 to evaluate the general health status of the animals. All carcinogen-exposed groups exhibited a reduction in body weight up to week 6 (Figure 7), which may be attributed to systemic stress, inflammatory responses, altered energy metabolism, and the early stages of tumor development induced by DMBA. This type of weight loss is commonly associated with carcinogen-induced cachexia and metabolic imbalance [41,42]. Importantly, no treatment was administered during this initial period; therefore, the observed weight loss reflects the direct impact of carcinogen exposure without therapeutic intervention.

Treatment started after week 6, and subsequent changes in body weight were indicative of the impact of the respective treatments. A slight increase in the body weight of the animals in the NC and VC groups was recorded after week 6. However, this is unlikely to be indicative of true physiological recovery and could be attributed to the progressive growth of tumors that contribute to overall body weight. The positive control group had a noticeable recovery in body weight, indicating a protective or therapeutic effect against carcinogen-induced systemic injury. The body weights of the FA gel- and KMP gel-treated groups gradually and consistently recovered after week 6, indicating partial alleviation of DMBA-induced toxicity and improvement in overall health status. The FA and KMP combination had the most consistent and noticeable increase in body weight, which was similar to that of the normal control group. This observation may indicate a synergistic effect of both drugs in mitigating carcinogen-induced physiological injury.

At the end of 16 weeks, significant tumor development was recorded in the negative control group, confirming the induction of tumors and progressive disease development. A similar pattern was observed in the vehicle control group, indicating that the vehicle did not exert any protective effect against tumor development. Compared with the negative control treatment, treatment with FA gel or KMP gel alone did not completely prevent tumor occurrence; however, both treatments reduced tumor multiplicity and overall tumor burden, suggesting moderate chemoprotective potential. The positive control group demonstrated a pronounced inhibitory effect on tumor progression, reflecting strong therapeutic efficacy. The combination therapy (FKMG) resulted in greater suppression of tumor development than the individual molecules did, indicating an enhanced or possible synergistic effect. Overall, while individual treatments provided partial protection, the combined formulation exhibited improved antitumor activity.

### 3.9. Histopathology

#### 3.9.1. Histopathological Evaluation of H&E-Stained Skin Sections

**Figure 8:**
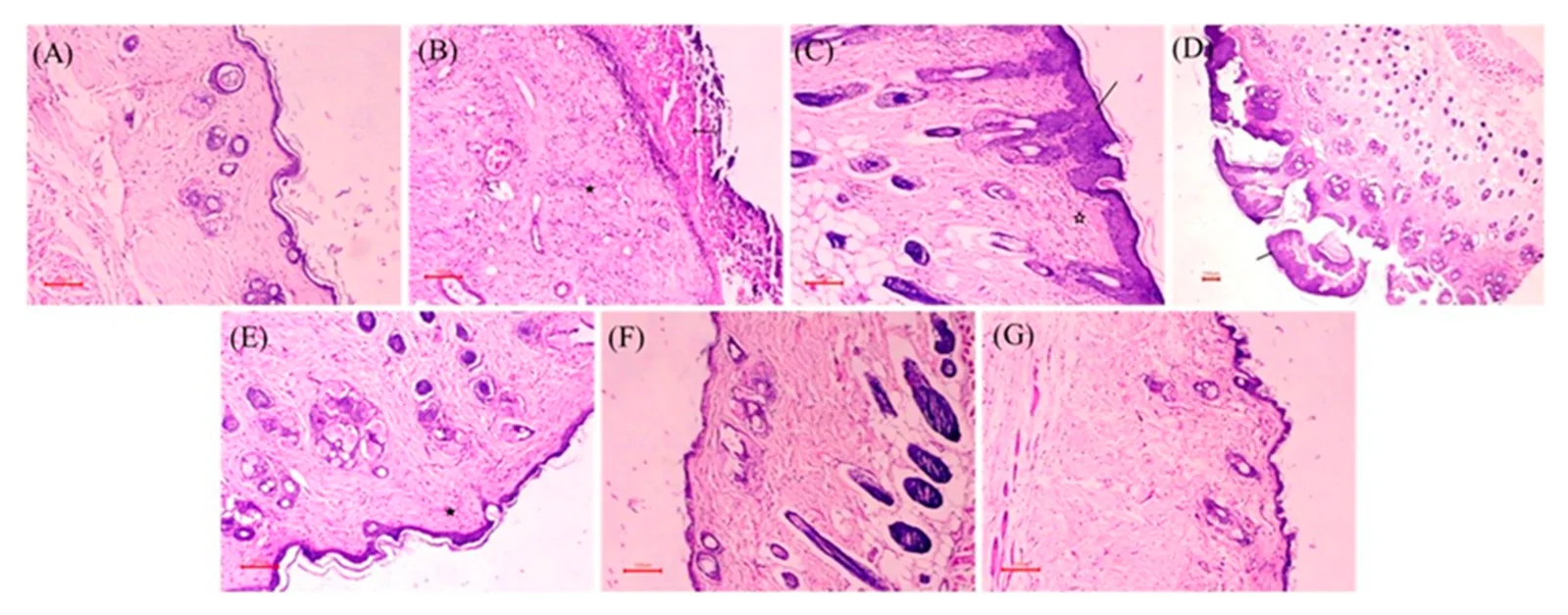
Histopathological features of the skin of a DMBA-induced mouse model: (**A**) control, (**B**) positive control, (**C**) negative control, (**D**) vehicle control, (**E**) KMPG-treated, (**F**) FAPG-treated, and (**G**) FKMG-treated groups. Scale 100 µm.

Histopathological examination of the skin tissues of all the experimental groups was performed to assess DMBA-induced skin damage and the protective efficacy of the test formulations. The normal control group exhibited intact skin architecture with a continuous epidermis, well-organized dermis, and normal collagen fiber distribution (Figure 8A). In contrast, the positive control group presented severe tissue injury characterized by complete loss of the epidermal layer, extensive inflammatory cell infiltration, necrotic debris, marked edema, hemorrhage, degeneration, and hyalinization of dermal collagen fibers, along with dilated blood vessels, reflecting treatment-associated cytotoxic effects (Figure 8B). The negative control group demonstrated characteristic DMBA-induced pathological changes, including moderate-to-severe epidermal hyperplasia, mild hyperkeratosis, inflammatory cell infiltration, and dermal collagen degeneration, indicating carcinogen-driven disease progression (Figure 8C). The vehicle control group largely retained normal skin architecture but exhibited focal areas of moderate-to-severe epidermal hyperplasia, mild hyperkeratosis, inflammation, and collagen fiber degeneration, suggesting minimal protective effects of the vehicle (Figure 8D). Treatment with KMPG resulted in a predominantly preserved skin structure with only mild-to-moderate collagen degeneration, indicating partial protection against DMBA-induced damage (Figure 8E). The FAPG-treated group presented largely normal skin parenchyma with occasional focal mild acanthosis and orthokeratotic hyperkeratosis, reflecting substantial restoration of epidermal and dermal integrity (Figure 8F). The FKMG-treated group exhibited nearly complete normalization of the skin histoarchitecture, closely resembling that of the normal control, and demonstrated superior protective efficacy among the treated groups (Figure 8G). Overall, these findings indicate that FKMG, followed by FAPG and KMPG, effectively mitigated DMBA-induced histopathological alterations and preserved skin tissue integrity.

#### 3.9.2. Histopathological Evaluation of H&E-Stained Spleen Sections

**Figure 9:**
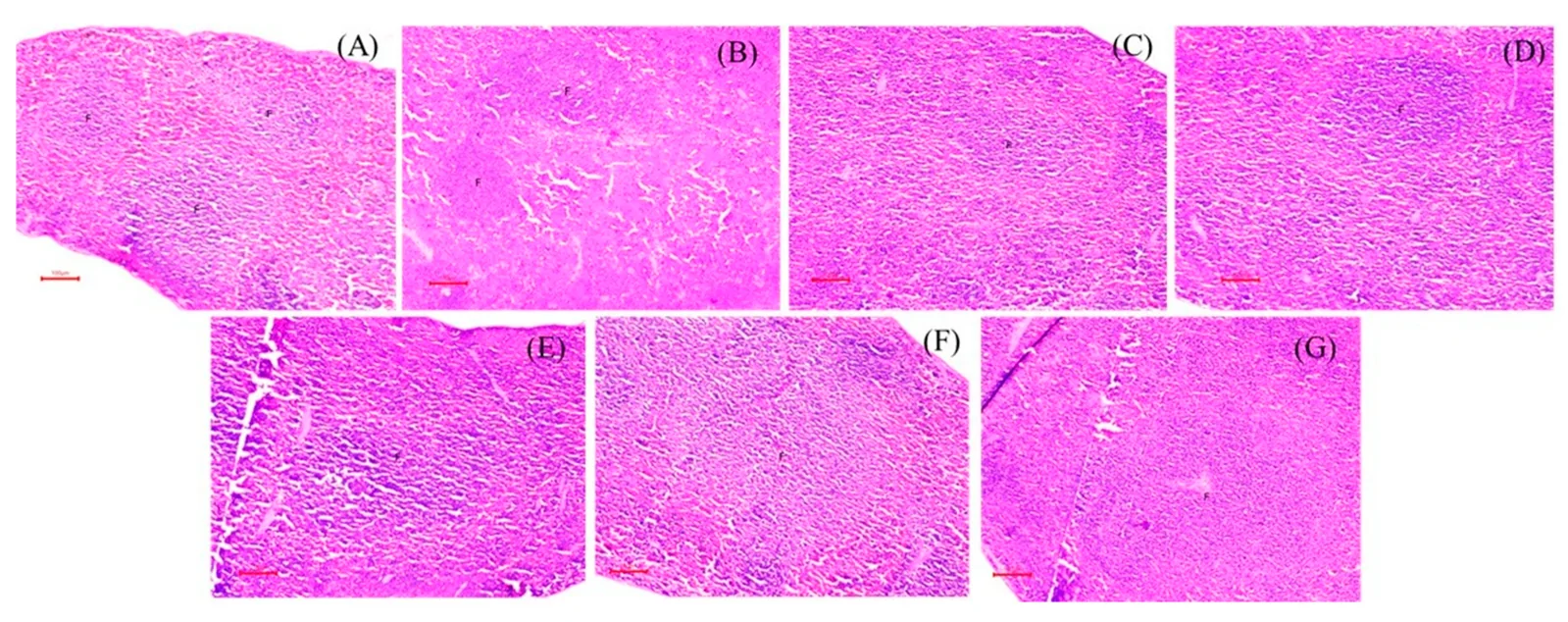
Histopathological features of spleen tissue in a DMBA-induced mouse model: control (**A**), positive control (**B**), negative control (**C**), vehicle control (**D**), KMPG-treated (**E**), FAPG-treated (**F**), and FKMG-treated groups (**G**). Scale 100 µm.

Histopathological examination of spleen sections was carried out to evaluate the systemic effects of DMBA exposure and the safety of the test formulations. The normal control group presented intact splenic parenchyma with well-preserved architecture, indicating normal spleen histology (Figure 9A). In comparison, the positive control, negative control, and vehicle control groups presented largely normal splenic architecture with only mild degenerative changes, suggesting minimal systemic involvement following topical DMBA application (Figure 9B–D). Treatment with FAPG resulted in normal splenic parenchyma with maintained structural integrity (Figure 9F). The KMPG-treated group also presented preserved splenic architecture without notable pathological alterations (Figure 9E). FKMG-treated group presented a splenic histology comparable to that of the normal control group, further confirming the systemic safety of the formulation (Figure 9G). Overall, these findings indicate that neither DMBA exposure nor treatment with the test formulations caused significant adverse histopathological changes in the spleen. This is likely due to the localized nature of the DMBA-induced skin carcinogenesis model, in which topical application limits systemic exposure, allowing secondary organs such as the spleen to retain normal architecture.

#### 3.9.3. Histopathological Evaluation of H&E-Stained Tumor Sections

**Figure 10:**
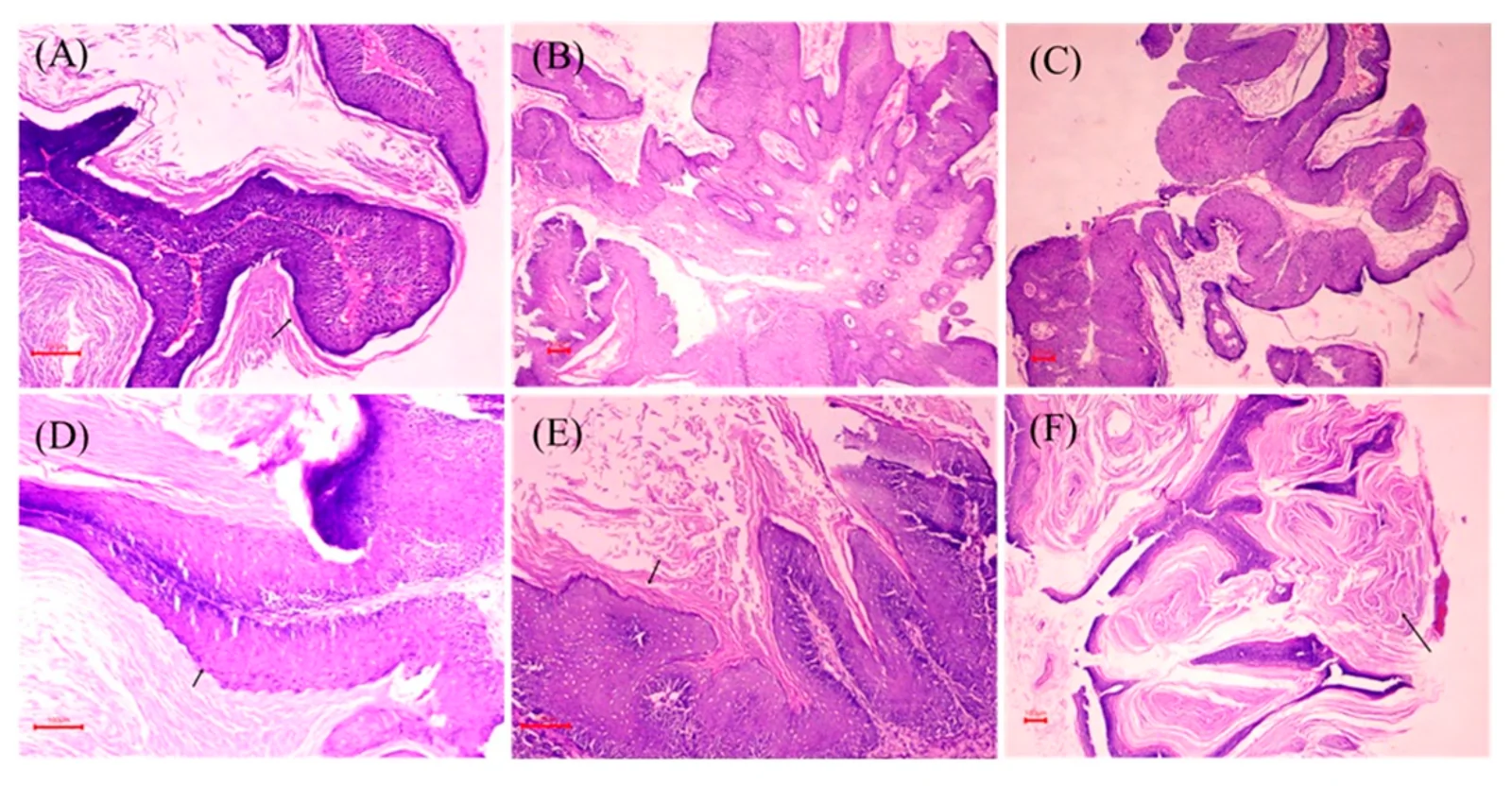
Histopathological features of tumor tissue from a DMBA-induced mouse model: positive control (**A**), negative control (**B**), vehicle control (**C**), KMPG-treated (**D**), FAPG-treated (**E**), and FKMG-treated (**F**) groups. Scale 100 µm.

Histopathological analysis of tumor sections after 16 weeks revealed marked differences among the experimental groups (Figure 10A–F). The positive control group revealed epidermal acanthosis with papillary projections, accompanied by mild-to-moderate parakeratosis–hyperkeratosis and fibrous stromal tissue, indicating partial suppression of DMBA-induced tumor progression. In contrast, the negative control group exhibited prominent papillary projections supported by thin fibrovascular cores, with epithelial papillary fronds lined by variably thick parakeratotic cells and abundant keratin material. These features were associated with marked acanthosis, combining parakeratotic and orthokeratotic hyperkeratosis, basal cell hyperplasia, sebaceous unit hyperplasia, and moderate inflammatory infiltration, confirming active tumor development. Compared with the negative control group, the vehicle control group presented histopathological features, including papillary projections, acanthosis, hyperkeratosis, basal cell hyperplasia, and mild inflammatory infiltration, indicating that the vehicle had no protective effect. Treatment with FAPG resulted in epidermal acanthosis with papillary projections and severe parakeratosis–hyperkeratosis, although with comparatively reduced epithelial proliferation. The KMPG-treated group presented similar architectural features but with better organization of the epidermal layers and reduced cellular atypia. FKMG-treated group exhibited pronounced keratinization with markedly reduced viable epithelial proliferation and a fibrous stromal background, suggesting the most effective therapeutic response among the treated groups.

### 3.10. Effects on the Weight and Size of the Spleen

**Figure 11:**
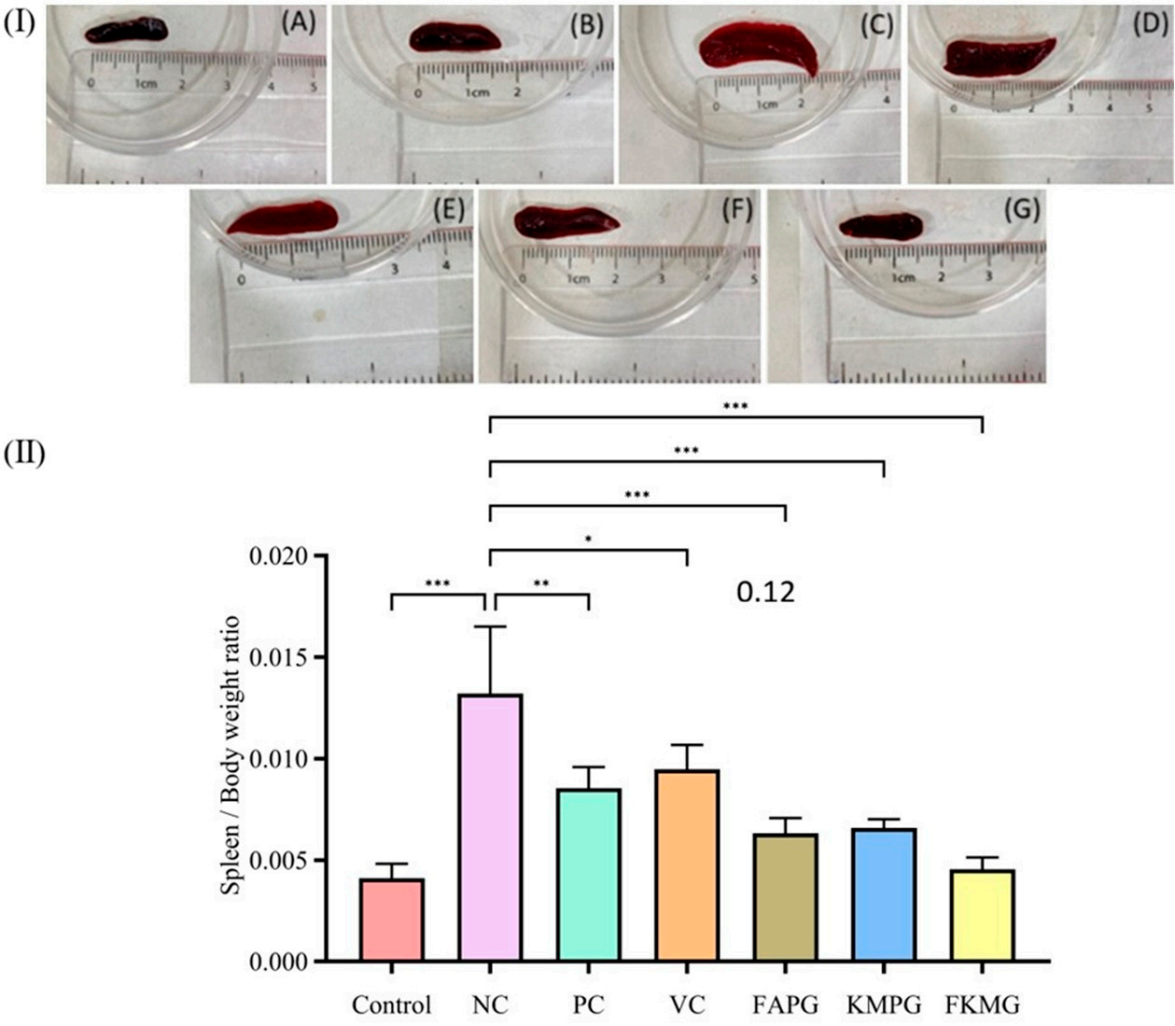
(**I**) Representative images illustrating the effects of treatment on spleen length ((**A**) control, (**B**) positive control, (**C**) negative control, (**D**) vehicle control, (**E**) KMPG-treated, (**F**) FAPG-treated, and (**G**) FKMG-treated), and (**II**) the spleen-to-body weight ratio in DMBA-induced Swiss albino mice (mean ± SD for n = 5). Statistical analysis was performed via one-way ANOVA with Dunnett’s multiple comparison test. The NC group was used as the reference for comparison (ns, *p* ≥ 0.12; * *p* < 0.033; ** *p* < 0.002; *** *p* < 0.001).

The spleen, the largest secondary lymphoid organ, performs a variety of immunological functions in addition to its functions in hematopoiesis and the clearance of red blood cells [43]. The average spleen in the human body is approximately 10 cm in length and weighs 150 g. However, in pathological cases, the spleen may weigh more than 2000 g and measure up to 30 cm in length. Spleen enlargement is referred to as splenomegaly. Splenomegaly may occur because of stress or because it is a chronic condition [44]. Figure 11 shows the spleen images and quantitative spleen-to-body weight ratio values of the DMBA-induced Swiss albino mice. The normal control group presented a physiological spleen/body weight ratio (0.0041 ± 0.0007), indicating normal splenic architecture. The positive control group presented an increase in spleen size with an increased spleen/body weight ratio (0.008 ± 0.001), reflecting partial persistence of DMBA-induced splenomegaly. In contrast, the negative control group demonstrated the greatest degree of spleen enlargement, with a markedly increased spleen/body weight ratio (0.0132 ± 0.003), confirming severe DMBA-induced splenic toxicity. Similarly, the vehicle control group exhibited pronounced splenomegaly (0.0094 ± 0.001), indicating that the vehicle alone failed to provide protection. Compared with the vehicle control, treatment with FAPG (0.0063 ± 0.0007), KMPG (0.0066 ± 0.0004), or FKMG (0.004 ± 0.0005) significantly reduced the spleen/body weight ratio. FKMG had the strongest protective effect, with values closely comparable to those of the normal control group, indicating effective attenuation of DMBA-induced splenomegaly and suggesting potent protective and immunomodulatory activity.

### 3.11. Effects on Biochemical Parameters

The metabolism of DMBA leads to the formation of reactive oxygen species (ROS) at the site of application, which may migrate to neighboring cells during tumorigenesis. Additionally, the persistent presence of tumor promoters leads to the formation of free radicals, which induce oxidative stress. Free radicals may cause damage to DNA. In conclusion, the production of free radicals and ROS is crucial in tumorigenesis and skin tumor promotion by DMBA and croton oil [45].

#### 3.11.1. Estimation of Glutathione S-Transferase Activity

**Figure 12:**
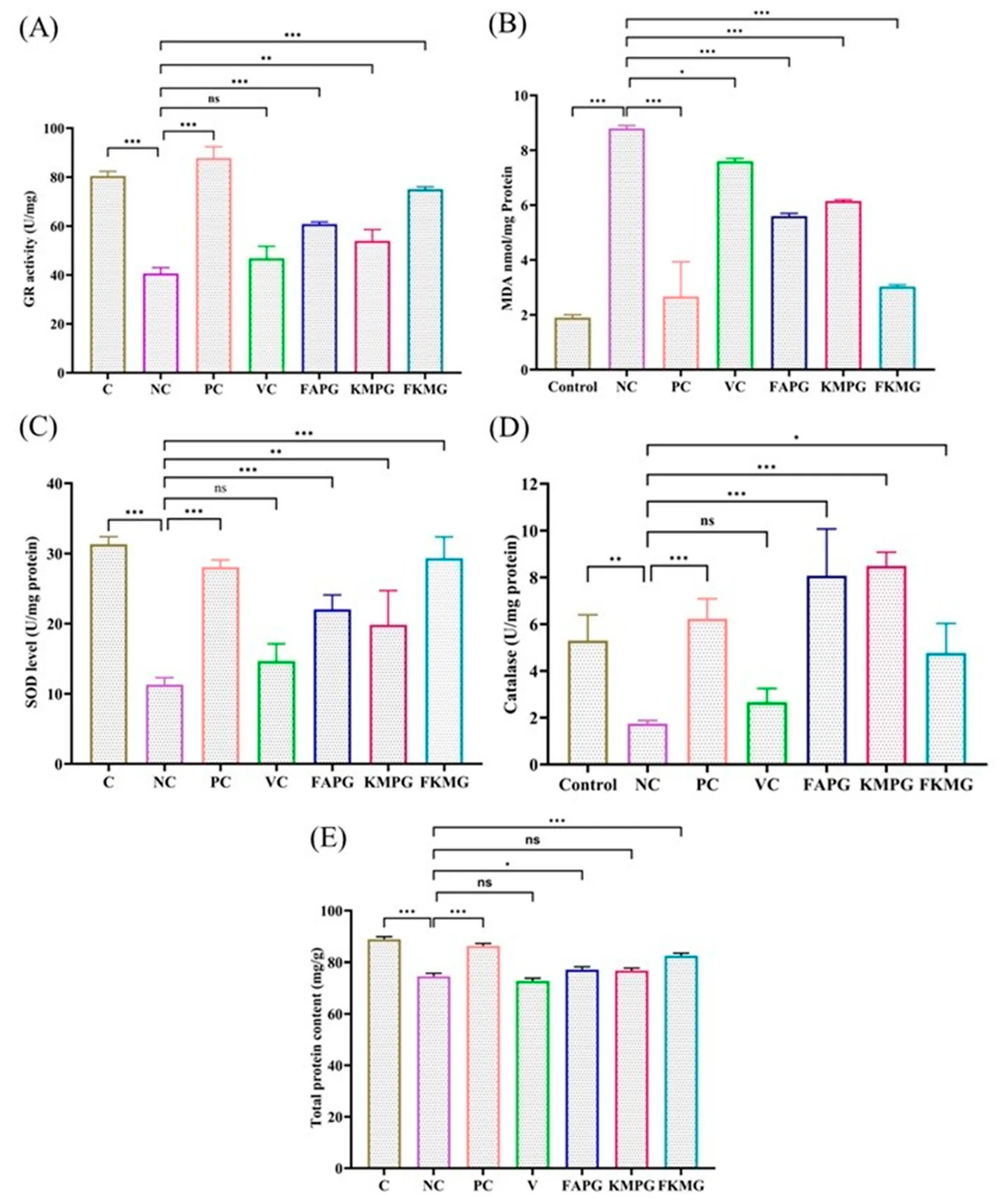
Effects of different treatments on (**A**) glutathione reductase activity, (**B**) lipid peroxidation activity, (**C**) superoxide dismutase activity, (**D**) catalase activity, and (**E**) total protein content (mean ± SD for n = 5). Statistical analysis was performed via one-way ANOVA with Dunnett’s multiple comparison test. The NC group was used as the reference for comparison (ns, *p* ≥ 0.12; * *p* < 0.033; ** *p* < 0.002; *** *p* < 0.001).

GSH is a nonenzymatic antioxidant tripeptide with one cysteine residue. It plays a crucial role in protecting cells from ROS and free radicals and affects the activity of lipoxygenase and cyclooxygenase enzymes in tumorigenesis [46]. Reduced glutathione assists in the detoxification of various toxic metabolites. Hence, when the levels of reduced glutathione are depleted in the cell, ROS overproduce, and oxidative stress is induced. Oxidative stress can affect the function and structure of membranes in cells and organelles [47]. Glutathione reductase activity was also determined to assess the level of modification of the antioxidant mechanism. The results for glutathione reductase are expressed as the fold change in comparison with the control group (Figure 12A). The negative control (NC) group showed a significant decrease, 0.48-fold greater than that of the control, indicating severe oxidative injury. The vehicle (V) group also presented a decrease of 0.65-fold. On the other hand, that of the positive control (PC) group increased 1.13-fold, suggesting enhanced antioxidant uptake. Among the treatment groups, FAPG and KMPG showed partial recovery, 0.76-fold and 0.71-fold greater than that of the control, respectively. The FKMG group showed a significant increase (0.94-fold) over the control level and was almost normal (Table 5).

#### 3.11.2. Estimation of Lipid Peroxidation

Lipid peroxidation is a normal process that is initiated by free radicals that participate in a chain reaction. Malondialdehyde, a lipid electrophile, is an important product of lipid peroxidation. An increase in malondialdehyde levels is an alarming sign associated with cancer [48]. In the present study, the normal control remained unchanged, indicating a normal, unstressed condition (Figure 12B). Compared with the normal control, the negative control and vehicle control significantly increased LPO by 4.63-fold and 4.00-fold, respectively, indicating extreme oxidative stress and damage to the membrane. Among the treatment groups, FAPG and KMPG caused moderate increases of 2.95-fold and 3.24-fold, respectively, indicating partial protection against oxidative stress. The formulation FKMG caused a smaller increase of 1.59-fold, and the positive control group only attained a 1.40-fold increase (Table 5). These results indicate that oxidative stress significantly increased lipid peroxidation and that treatment with various compounds, particularly FKMG, effectively reduced MDA production and protected membrane lipids against oxidative damage.

#### 3.11.3. Estimation of Superoxide Dismutase Activity

Superoxide dismutase (SOD), an important antioxidant enzyme that scavenges superoxide radicals, was analyzed to determine the level of oxidative stress and antioxidant activity. SOD catalyzes the reaction of the superoxide anion to form hydrogen peroxide and molecular oxygen, thus protecting cells from oxidative stress. SOD activity is an important indicator of the body’s ability to neutralize ROS [49]. In the present study, the obtained values are presented as the fold change compared with the control group (31.33 U/mg protein) (Figure 12C). SOD activity significantly decreased in the NC (0.36-fold) and VC (0.47-fold) groups, which indicated a substantial level of oxidative stress compared with that in the normal control group. However, the positive control group presented a significant improvement in SOD activity, reaching 0.90-fold greater than the control value, which indicated a substantial reduction in oxidative stress. In the treatment groups, FAPG and KMPG moderately improved SOD activity, reaching 0.70-fold and 0.63-fold greater, respectively, than the control values, which indicated a partial improvement in antioxidant activity (Table 5). Importantly, the FKMG group presented the greatest improvement among the treatment groups, reaching 0.94-fold greater than the control value, which was remarkably close to the normal physiological level.

#### 3.11.4. Estimation of Catalase Activity

Catalase (CAT) is a very important part of the cell defense system. It helps reduce the oxidative stress caused by ROS. Among enzymes, catalase has the highest catalytic efficiency. A single catalase molecule can oxidize millions of H_2_O_2_ molecules to water and oxygen within a second. The high catalytic efficiency of this enzyme emphasizes its role in protecting cells from oxidative stress. Catalase can modulate apoptosis because it can influence the redox state, which increases the sensitivity of cells to apoptosis. Unregulated catalase activity has been shown to cause an imbalance in the apoptotic pathway [50]. The catalase activity was assessed in all the experimental groups. The control group presented normal basal levels of catalase activity (Figure 12D). The negative control group (0.33-fold) and the vehicle control group (0.50-fold) presented significantly lower catalase activity than the control. Compared with the control group, the positive control group presented a slight increase (1.18-fold) in catalase activity. Among the experimental groups, the catalase activity of FAPG (1.52-fold) and KMPG (1.60-fold) increased, whereas that of FKMG increased 0.90-fold (Table 5). Importantly, among all the experimental groups, the FKMG group presented the greatest degree of catalase activity, which was closest to that of the control group; thus, among all the experimental groups, the FKMG group presented better normalization of catalase activity.

#### 3.11.5. Estimation of Total Protein Content

The total protein content was evaluated in all the experimental groups (n = 5). The control group had a total protein level of 88.87 ± 1.00 mg/g, which is a normal physiological level of protein. Compared with the control group, the negative control (NC) group presented a significantly lower total protein level (74.63 ± 1.15 mg/g). The vehicle group presented lower protein levels (72.80 ± 1.05 mg/g). However, the positive control (PC) group presented a greater protein level (86.30 ± 0.95 mg/g), which was similar to that of the control group. Compared with those in the NC and vehicle groups, the total protein levels in the FAPG (77.20 ± 1.10 mg/g) and KMPG (76.87 ± 0.95 mg/g) treatment groups moderately improved. Compared with the control group, the FKMG group presented a greater protein level (82.43 ± 1.05 mg/g), which was closest to that of the control group among all the treatment groups (Figure 12E and Table 5). In general, these results suggest that treatment, especially FKMG, effectively increased total protein levels to near-normal physiological levels.

### 3.12. Proinflammatory Cytokines

**Figure 13:**
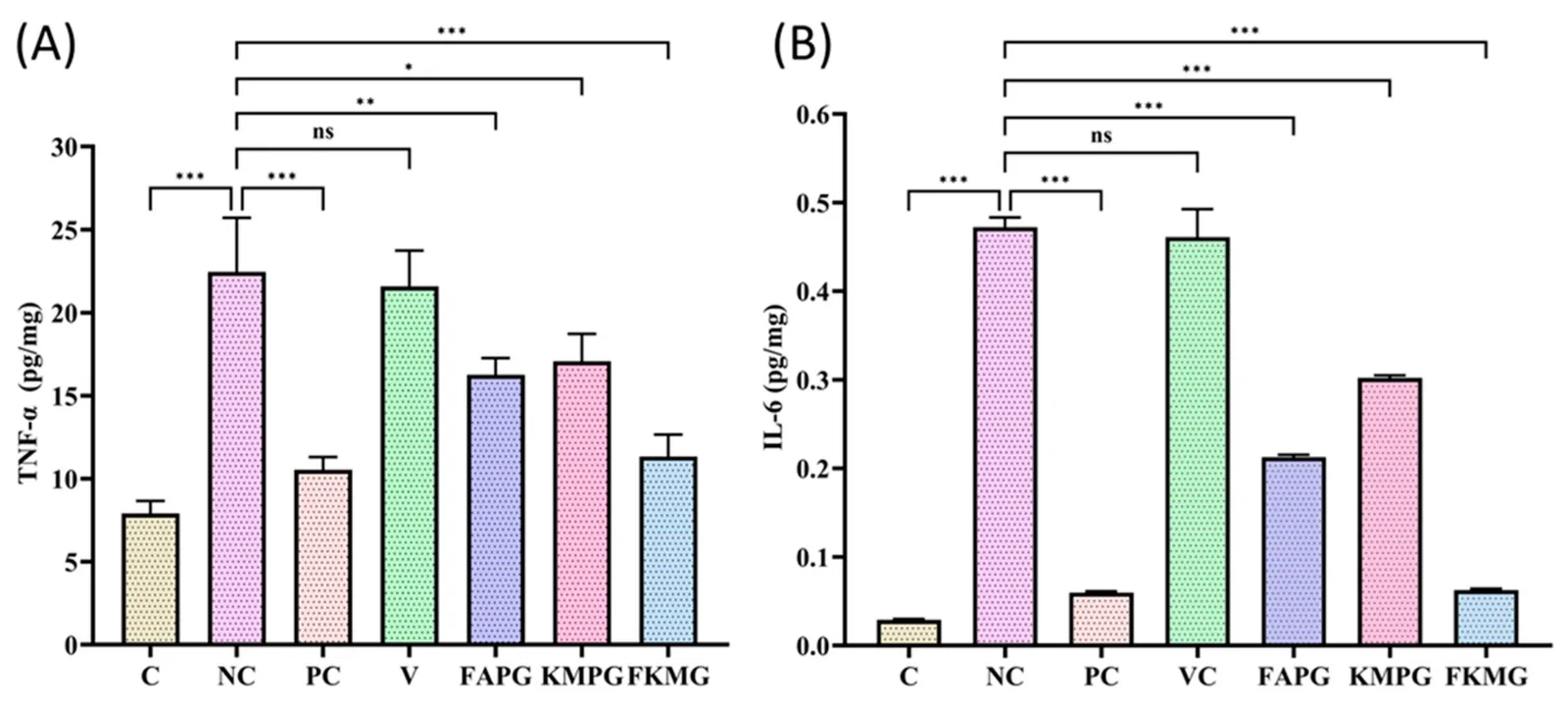
Effects of treatment on TNF-α levels (**A**) and IL-6 levels (**B**) in DMBA-induced Swiss albino mice (mean ± SD for n = 5). Statistical analysis was performed via one-way ANOVA with Dunnett’s multiple comparison test. The NC group was used as the reference for comparison (ns, *p* ≥ 0.12; * *p* < 0.033; ** *p* < 0.002; *** *p* < 0.001).

TNF-α is a proinflammatory cytokine that has a role in tumor development. In the DMBA carcinogenesis process, TNF-α increases the nuclear translocation of NF-κB (a transcription factor), which promotes the survival and growth of neoplastic cells [51]. The inflammatory response in each experimental group was assessed by measuring TNF-α levels. There were notable differences in the TNF-α levels among the groups (Figure 13A). The TNF-α level was significantly greater in the DMBA-induced untreated group (NC), at 22.45 ± 3.26 pg/mg, indicating an enhanced inflammatory response after DMBA exposure, than in the normal control (C) group, at 7.90 ± 0.74 pg/mg. The TNF-α level in the vehicle (VC) group was 21.58 ± 2.11 pg/mg, which was similar to that in the NC group, with a nonsignificant difference. These findings suggest that the vehicle itself did not affect the inflammatory response. The TNF-α level considerably decreased to 10.53 ± 0.73 pg/mg in the positive control (PC), with values that were comparable to those of the normal control group. The TNF-α levels in the FAPG and KMPG groups in the experimental groups were 16.25 ± 1.00 pg/mg and 17.08 ± 1.75 pg/mg, respectively, indicating moderate decreases compared with those in the NC group. FKMG therapy significantly reduced TNF-α levels to 11.35 ± 1.30 pg/mg, suggesting a significant reduction in inflammation caused by DMBA. The TNF-α levels were greater after DMBA exposure, but they were approximately normal control levels after PC and FKMG treatments.

IL-6, an essential proinflammatory and signaling messenger, is crucial for the promotion of carcinogenesis. Higher IL-6 production has been linked to the promotion phase of skin carcinogenesis, and this increase is consistent with earlier findings showing that DMBA/croton oil-induced skin carcinogenesis stimulates excessive IL-6 production, which is essential for tumor promotion and STAT3 signaling pathway activation [52]. Figure 13B shows the effects of the various treatments on the IL-6 levels in the DMBA/croton oil-induced experimental animals. The IL-6 level decreased in the FKMG (0.063 ± 0.002) and positive control (PC) groups (0.060 ± 0.002), whereas KMPG (0.302 ± 0.003) and FAPG (0.213 ± 0.003) caused moderate inhibition compared with the NC group. The drastic decrease in IL-6 levels in FKMG indicates its potent anti-inflammatory and probable chemopreventive properties, which may be attributed to the inhibition of proinflammatory signaling pathways. Collectively, these findings indicate that attenuation of IL-6 production may be a key mechanism underlying the protective effects of the tested formulations against DMBA/croton oil-induced inflammation and tumor promotion.

### 3.13. Stability Study

The optimized NPs exhibited good physicochemical stability during 6 months of storage (Supplementary Figure S1).

## 4. Conclusions

FA and KMP-loaded hydrogels demonstrated synergistic effects in the treatment of skin cancer. The formulated NP hydrogel system showed high viscosity and good spreadability, making it suitable for topical application. In vitro release studies confirmed sustained drug release. In vivo studies in Swiss albino mice revealed that topical application of the FA-KMP hydrogel (FKMG) significantly suppressed DMBA/croton oil-induced skin carcinogenesis, as evidenced by a reduction in tumor number and incidence, which was supported by histopathological findings. The formulation also protects against oxidative stress by increasing antioxidant defense enzyme activity and significantly reducing lipid peroxidation levels. Furthermore, it effectively modulated proinflammatory cytokines such as TNF-α and IL-6. Although formulation FKMG did not demonstrate greater antitumor efficacy than 1% Flonida^®^ cream for every evaluated parameter, its therapeutic action differs fundamentally from that of 5-fluorouracil. While 5-fluorouracil primarily produces its effect through direct cytotoxicity against rapidly proliferating cells, FKMG acts through multiple complementary mechanisms, including sustained local drug delivery, reduction in oxidative stress, suppression of chronic inflammation, restoration of endogenous antioxidant defence, and preservation of normal tissue architecture. This multimodal mode of action may offer an advantage for long-term chemoprevention by simultaneously limiting tumor progression while maintaining the integrity of healthy skin. Overall, the results of this study suggest that a hydrogel containing FA and KMP may have great potential for preventing and treating skin cancer.

## Figures and Tables

**Table 1:** Animal groups and treatments given to male Swiss albino mice.

Experimental Groups	Treatment
Group I (Normal)	Normal food and water
Group II (Negative control)	DMBA + 1% croton oil
Group III (Positive control)	DMBA + 1% Flonida cream + 1% croton oil
Group IV (Vehicle control)	DMBA + 2% HA + 1% croton oil
Group V (FAPG)	DMBA + FAPG (FA equivalent to 45 mg/kg/day) + 1% croton oil
Group VI (KMPG)	DMBA + KMPG (KMP equivalent to 30 mg/kg/day) + 1% croton oil
Group VII (FKMG)	DMBA + FKMG (equivalent to FA of 45 mg/kg/day and KMP of 30 mg/kg/day) + 1% croton oil

**Table 2:** Results of Bingham plastic, power-law, and Herschel–Bulkley models on rheological data of hydrogel formulations.

Formulation	Model	τ_0_ (Pa)	k (Pa·s^n^)	n	ηB (Pa·s)	R^2^
PPG	Bingham	81.93	--	--	0.1941	0.8851
	Power Law	--	3.17	0.6637	--	0.9196
	Herschel–Bulkley	32.81	3.27	0.6225	--	0.9570
PNG	Bingham	52.39	--	--	0.1457	0.8963
	Power Law	--	2.2	0.6647	--	0.9563
	Herschel–Bulkley	17.71	3.2	0.5771	--	0.9717
FAPG	Bingham	103.95	--	--	0.2181	0.8732
	Power Law	--	4.23	0.6455	--	0.8999
	Herschel–Bulkley	42.91	6.46	0.5385	--	0.9766
KMPG	Bingham	110.53	--	--	0.2285	0.8695
	Power Law	--	4.05	0.6614	--	0.8876
	Herschel–Bulkley	45.62	8.18	0.5094	--	0.9830
FKMG	Bingham	232.38	--	--	0.3593	0.8187
	Power Law	--	17	0.524	--	0.9023
	Herschel–Bulkley	110.76	20	0.45	--	0.9798

**Table 3:** Results of kinetic analysis of drug release data.

Formulation	Drug	Zero Order (r^2^)	First Order (r^2^)	Higuchi Model (r^2^)	Korsmeyer-Peppas Model (r^2^)	n Value in Korsmeyer-Peppas Model
FKMG	FA	0.9008	0.9318	0.9681	0.9747	0.63
	KMP	0.8858	0.9416	0.9557	0.9685	0.71
FAPG	FA	0.8746	0.9512	0.9799	0.9803	0.58
KMPG	KMP	0.903	0.9735	0.9749	0.9893	0.70

**Table 4:** Effects of various treatments on body weight, tumor incidence, and tumor burden in DMBA-croton oil-induced Swiss albino mice (16 weeks).

Group	Weight (g)			Tumor Incidence (%)	Tumor Burden
	Initial Weight (Week 1)	Final Weight (Week 16)	Net Weight Gain (%)		
Group I (Normal)	31.2 ± 0.32	42.6 ± 0.58	36.5 ± 0.6	0%	0
Group II (Negative control)	33.8 ± 0.35	36.9 ± 0.62	9.2 ± 1.1	100%	12.8
Group III (Positive control)	30.9 ± 0.28	37.8 ± 0.55	22.3 ± 0.9	20%	2.0
Group IV (Vehicle control)	34.5 ± 0.40	38.9 ± 0.60	12.8 ± 1.0	100%	10.0
Group V (FAPG)	32.6 ± 0.30	39.8 ± 0.57	22.1 ± 0.7	100%	6.4
Group VI (KMPG)	31.8 ± 0.29	38.6 ± 0.53	21.4 ± 0.8	100%	8.0
Group VII (FKMG)	34.1 ± 0.33	44.4 ± 0.65	30.2 ± 0.6	60%	4.67

**Table 5:** Biochemical alterations in oxidative stress and antioxidant parameters following topical treatment in the DMBA-induced skin tumor model.

Group	Catalase (U/mg)	LPO (nmol/mg Protein)	Total Protein (mg/g)	SOD (U/mg)	GSH (U/mg)
Group I (Normal)	5.30 ± 1.10	1.90 ± 0.10	88.87 ± 1.00	31.33 ± 1.04	79.76 ± 0.77
Group II (Negative control)	1.75 ± 0.13	8.80 ± 0.10	74.63 ± 1.15	11.30 ± 1.00	38.63 ± 0.63
Group III (Positive control)	6.23 ± 0.36	2.67 ± 1.27	86.30 ± 0.95	28.07 ± 1.01	90.12 ± 0.97
Group IV (Vehicle control)	2.67 ± 0.58	7.60 ± 0.10	72.80 ± 1.05	14.67 ± 2.48	51.79 ± 0.71
Group V (FAPG)	8.07 ± 2.00	5.60 ± 0.10	77.20 ± 1.10	22.03 ± 2.05	60.73 ± 0.70
Group VI (KMPG)	8.49 ± 0.59	6.15 ± 0.05	76.87 ± 0.95	19.83 ± 4.88	57.03 ± 0.62
Group VII (FKMG)	4.77 ± 1.27	3.03 ± 0.06	82.43 ± 1.05	29.33 ± 3.06	75.11 ± 0.75

## Data Availability

The original contributions presented in this study are included in the article/Supplementary Material. Further inquiries can be directed to the corresponding author.

## Supplementary Materials

The following supporting information can be downloaded at https://www.mdpi.com/article/10.3390/pharmaceutics18080972/s1, Figure S1: Stability evaluation of optimized PLGA nanoparticles after 6 months of storage. Particle size distribution following storage at (A) 25 ± 2 °C/60 ± 5% RH and (B) 5 ± 3 °C, and zeta potential distribution following storage at (C) 5 ± 3 °C and (D) 25 ± 2 °C/60 ± 5% RH.

## Abbreviations

The following abbreviations are used in this manuscript:

DMBA	7,12-Dimethylbenz[a]anthracene
TNF-α	Tumor Necrosis Factor-alpha
IL-6	Interleukin-6
PI3K/Akt	Phosphoinositide 3-Kinase/Protein Kinase B (Akt) Pathway
PLGA	Poly(lactic-co-glycolic acid)
DNA	Deoxyribonucleic Acid
DTNB	5,5′-Dithiobis(2-nitrobenzoic acid)
NADPH	Nicotinamide Adenine Dinucleotide Phosphate
MDA	Malondialdehyde
TBARS	Thiobarbituric Acid-Reactive Substances
ANOVA	Analysis of Variance
ROS	Reactive Oxygen Species
