# Supplementary material for: Nanoenabled Hyaluronic Acid-Based Topical Delivery of Kaempferol and Ferulic Acid: Chemomodulatory Potential Against DMBA-Croton Oil-Induced Skin Carcinogenesis in Male Swiss Albino Mice
Source: Pharmaceutics. 2026 Aug 7;18(8):972. doi: 10.3390/pharmaceutics18080972 (PMC13516110; doi:10.3390/pharmaceutics18080972)
Supplement: Supplementary file 1 [file pharmaceutics-18-00972-s001.zip › pharmaceutics-4407311-supplementary.pdf]

Nanoenabled Hyaluronic Acid-Based Topical Delivery of Kaempferol and Ferulic Acid: Chemomodulatory Potential Against DMBA-Croton Oil-Induced Skin Carcinogenesis in Male Swiss Albino Mice

Moulika Todaria [1], and Rajendra Awasthi [2,*]

Stability of Optimized PLGA Nanoparticles

The physical stability of the optimized PLGA nanoparticles was evaluated after storage for 6 months under refrigerated conditions (5 ± 3 °C) and ambient conditions (25 ± 2 °C/60 ± 5% RH). Particle size distribution and zeta potential were analyzed to determine any changes in the physicochemical characteristics of the nanoparticles.

As shown in Figures S1A and S1B, the optimized nanoparticles maintained a narrow and unimodal particle size distribution after 6 months of storage under both conditions. The mean particle size was 195.1 nm with a PDI of 0.207 following storage at 5 ± 1 °C, whereas nanoparticles stored at 25 ± 2 °C/60 ± 5% RH exhibited a mean particle size of 197.9 nm with a PDI of 0.251. The slight variation in particle size and polydispersity index was negligible, indicating the absence of significant particle aggregation or instability during the storage period. Although a minor secondary peak with very low intensity (~1.9%) was observed in samples stored under refrigerated conditions, the primary population accounted for more than 98% of the total intensity, confirming that the nanoparticles predominantly retained their original size distribution. The zeta potential values obtained after storage (-12.26 and -9.675 mV at 5 ± 1 °C and 25 ± 2 °C, respectively) are presented in Figure S1C and S1D. The electrophoretic mobility profiles remained unimodal under both storage conditions with no noticeable shift in the peak position, indicating that the surface charge of the nanoparticles was largely preserved throughout the storage period. The absence of significant changes in zeta potential suggests that electrostatic stabilization of the nanoparticles was maintained, thereby minimizing particle aggregation during storage. Overall, the stability data demonstrates that the optimized PLGA nanoparticles retained their physicochemical characteristics after 6 months under both refrigerated and ambient storage conditions. The minimal changes in particle size distribution, PDI, and zeta potential indicate good colloidal stability of the optimized formulation, supporting its suitability for further incorporation into the hydrogel matrix.

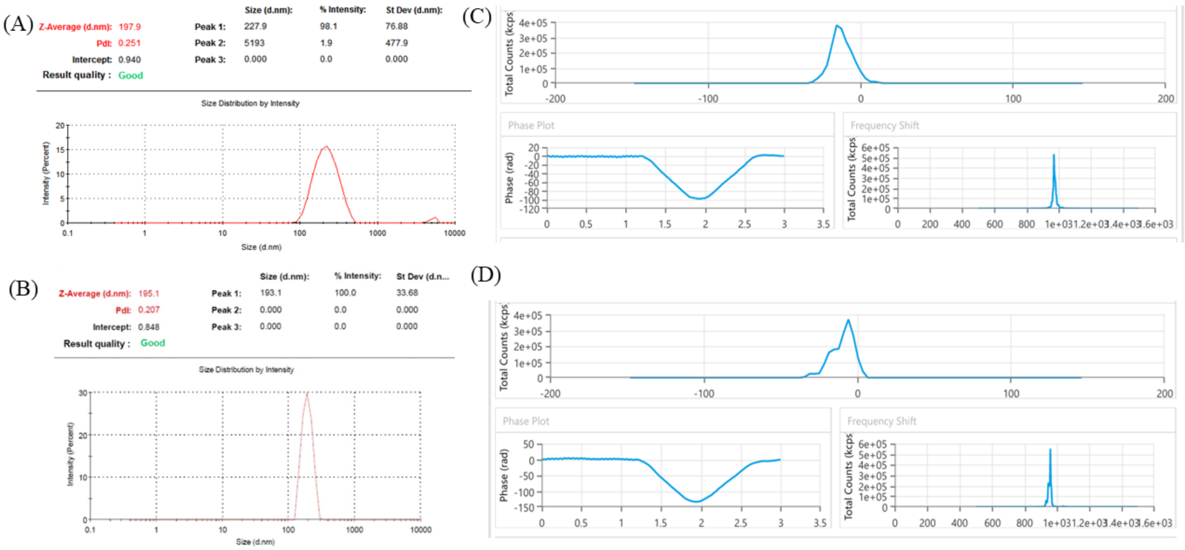

Figure S1. Stability evaluation of optimized PLGA nanoparticles after 6 months of storage. Particle size distribution following storage at (A) 25 ± 2 °C/60 ± 5% RH and (B) 5 ± 3 °C, and zeta potential distribution following storage at (C) 5 ± 3 °C and (D) 25 ± 2 °C/60 ± 5% RH.
